# A Mouse-Based Method to Monitor Wheat Allergens in Novel Wheat Lines and Varieties Created by Crossbreeding: Proof-of-Concept Using Durum and *A. tauschii* Wheats

**DOI:** 10.3390/ijms23126505

**Published:** 2022-06-10

**Authors:** Rick Jorgensen, Rajsri Raghunath, Haoran Gao, Eric Olson, Perry K. W. Ng, Venu Gangur

**Affiliations:** 1 Food Allergy & Immunology Laboratory, Department of Food Science & Human Nutrition, Michigan State University, East Lansing, MI 48824, USA; jorgen70@msu.edu (R.J.); raghun14@msu.edu (R.R.); gaohaora@msu.edu (H.G.); 2 Wheat Breeding & Genetics Laboratory, Department of Plant, Soil and Microbial Sciences, Michigan State University, East Lansing, MI 48824, USA; eolson@msu.edu; 3 Cereal Science Laboratory, Department of Food Science & Human Nutrition, Michigan State University, East Lansing, MI 48824, USA; ngp@msu.edu

**Keywords:** wheat allergy, wheat allergens, mouse model, anaphylaxis, wheat breeding, wheat genotype, *A. tauschii* wheat, durum wheat, western blotting, protein sequencing

## Abstract

Wheat allergies are potentially life-threatening because of the high risk of anaphylaxis. Wheats belong to four genotypes represented in thousands of lines and varieties. Monitoring changes to wheat allergens is critical to prevent inadvertent ntroduction of hyper-allergenic varieties via breeding. However, validated methods for this purpose are unavailable at present. As a proof-of-concept study, we tested the hypothesis that salt-soluble wheat allergens in our mouse model will be identical to those reported for humans. Groups of Balb/cJ mice were rendered allergic to durum wheat salt-soluble protein extract (SSPE). Using blood from allergic mice, a mini hyper-IgE plasma bank was created and used in optimizing an IgE Western blotting (IEWB) to identify IgE binding allergens. The LC-MS/MS was used to sequence the allergenic bands. An ancient *Aegilops tauschii* wheat was grown in our greenhouse and extracted SSPE. Using the optimized IEWB method followed by sequencing, the cross-reacting allergens in *A. tauschii* wheat were identified. Database analysis showed all but 2 of the durum wheat allergens and all *A. tauschii* wheat allergens identified in this model had been reported as human allergens. Thus, this model may be used to identify and monitor potential changes to salt-soluble wheat allergens caused by breeding.

## 1. Introduction

Wheat allergy is a major food safety issue that affects wheat products. Contamination with major food allergens including wheat is a leading cause of food recalls in the USA [1]. Prevalence of wheat allergies along with other major types of food allergies such as nut allergies, has been increasing not only in the USA but also in many other developed countries including Canada, Australia, Japan, European Union countries, and the United Kingdom [2,3,4,5]. Because food allergens including wheat can trigger life-threatening systemic anaphylaxis, they are a serious concern for the food industry, and public health [6,7]. Therefore, preventing inadvertent creation of hyper-allergenic wheats by wheat breeding is a major challenge facing wheat breeders.

Food allergies currently affect 8% of children and 10.8% of adults in the USA [6,8]. Sensitization to wheat (i.e., presence of IgE antibodies in the blood that bind to wheat proteins) in the United States is around 3.6% [9]. Clinically confirmed wheat allergies affect 0.4% of the population in the USA [10]; prevalence at the global level is estimated to be ~0.9% [3]; and affects both adults and children of both genders [2,11]. Thus, wheat allergy is a major growing global public health problem that must be addressed immediately.

There are two major types of immune system-mediated diseases caused by wheat consumption: (i) IgE antibody-mediated allergic diseases that include, classical food allergies (with symptoms of the gastrointestinal tract, and systemic anaphylaxis), atopic dermatitis, urticaria, baker’s asthma, and allergic rhino-conjunctivitis; and (ii) non-IgE-mediated immune diseases that include celiac disease (an autoimmune disease), non-celiac gluten sensitivity, and eosinophil-mediated diseases (e.g., eosinophilic esophagitis, eosinophilic gastroenteritis etc.) [12,13,14,15,16,17,18,19,20]. Notably, IgE-mediated allergic reactions such as systemic anaphylaxis and allergic asthma are potentially lethal [6,7]. Therefore, identification of specific wheat allergens associated with life-threatening diseases is a critical first step towards diagnosis and management of wheat allergies.

Wheat proteins are classified based on the solubility of the non-glutens (water/salt-soluble albumins/globulins) and the glutens (alcohol-soluble gliadins and acid-soluble glutenins) [21,22]. The non-glutens represent 15–20% of total proteins, while the glutens comprise the rest [23,24,25]. Notably, both types of wheat proteins can trigger IgE-mediated allergic reactions in humans and in animal models [2,11,22].

Wheat allergy develops in two phases: sensitization to wheat allergens, and wheat allergy disease elicitation in sensitized individuals [6,26,27]. Sensitization occurs when genetically susceptible subjects are exposed to wheat products via physiologic routes such as oral, nasal, dermal, or conjunctival thus commencing the production of IgE antibodies to wheat allergens, resulting in sensitization [6,22]. The second phase of development of a wheat allergy is characterized by disease elicitation upon re-exposure to wheat allergens. Re-exposure to wheat allergens in sensitized individuals results in the binding of allergens to the IgE now present on the mast cells and basophils that trigger release of histamine and other mediators causing disease symptoms including life-threatening anaphylaxis [11,22,26,28]. Therefore, it is possible to identify wheat allergens that cause allergic reactions based on their ability to bind to IgE antibodies obtained from wheat-allergic humans and animal models such as mice.

Wheat is among the top 3 cereals (the others being rice and corn) consumed worldwide as a staple food by billions of people [29]. Wheat production and consumption has been increasing worldwide in general. For example, wheat production has increased from 731.4 million tonnes in 2018, to 776.1 tonnes in 2020 [29]. However, recent data shows that while production is increasing, its consumption has taken a downward trend in the USA for reasons yet to be determined [30]. One potential reason for this trend may be the avoidance of wheat products due to real or perceived health concerns [31,32]. Therefore, identification and systematic monitoring of wheat allergens in various wheat varieties and wheat lines has become more urgent than ever before.

Commonly consumed wheats can be classified into three distinct genotypes, namely, AA, AABB, and AABBDD [21,28]. The ancient *A. tauschii* wheat of the DD genotype is not commercially available. The commonly consumed wheats produce wheat products meant for human and animal usage [28]. Using conventional cross-hybridization, and back-crossing, wheat breeders have produced thousands of wheat varieties and wheat lines primarily to enhance agronomical phenotypes and for enhancing profitability [33,34]. However, the impact of these changes on allergenicity properties of wheat such as composition of wheat allergens has not been well studied. A major reason for this is the unavailability of validated and reliable methods to monitor changes to wheat allergens resulting from wheat breeding. There are efforts to generate wheat lines lacking specific allergens such as ω-5 gliadin [35]. Although genetically modified (GM) wheat by recombinant DNA technology is not commercially available, future researchers may consider such an approach. An international expert panel organized by the FAO/WHO had previously provided a decision-tree framework for assessing allergenic potential of GM foods so that they could be compared to the native varieties in determining ‘substantial equivalence’ between the GM vs. non-GM varieties [36,37,38,39]. A similar approach may also be used to compare allergenicity of novel wheat varieties and lines developed by conventional wheat breeding with the currently established wheats. Currently, methods used to identify potential wheat allergens include using IgE from human allergic subjects and using database approaches by sequence comparisons. Use of appropriate animal models for pre-clinical assessment of novel foods has been suggested [28]. However, a fully validated animal model is not yet available although efforts are underway in that direction.

There are many animal models of wheat allergenicity including dogs, rats, and mice that have been valuable for studying mechanisms of wheat allergenicity [22,28,40]. Among these models, mice are most widely studied because of their short generation time, availability of gene knockout mice and reagents, and for economic reasons [41]. In addition, mouse models can also potentially serve as a valuable in vivo tool for monitoring changes to wheat protein allergenicity introduced by wheat breeding and food processing. However, the mouse models must be extensively validated to simulate human wheat allergenicity as closely as possible to demonstrate their power to predict human allergenicity hazards. We have previously shown that salt-soluble protein extract (SSPE) from durum wheat can be used to clinically sensitize mice for life-threatening systemic anaphylaxis [40,42]. However, it is unclear whether specific wheat allergens that elicit IgE antibodies and consequently cause sensitization for systemic analysis in this mouse model are identical to those that trigger human wheat allergies—the focus of this study. It is critical to validate this in this mouse model so that the model can be pro-actively applied to monitor changes to wheat allergens potentially due to wheat breeding.

Here, we tested the hypothesis that specific wheat allergens in our mouse model will be similar to human wheat allergens reported in the database. There were 4 objectives in this study: (i) to optimize and validate an IgE Western blot (IEWB) method using hyper IgE plasma obtained from durum wheat-allergic mice; (ii) to determine the IgE-binding protein bands in the IEWB followed by allergen identification by LC-MS/MS sequencing; (iii) to identify the cross-reacting allergens present in the ancient *A. tauschii* wheat using the IgE antibodies from the durum wheat-allergic mice; and (iv) to compare the mouse model wheat allergens to the human wheat allergens reported in the database. Our results collectively demonstrate that all, but two, of the allergens identified in this mouse model, are indeed reported as human wheat allergens. Therefore, these data provide the proof-of-concept that the mouse model may be used to identify and monitor changes to wheat allergens due to wheat breeding.

## 2. Results

### 2.1. Identification of IgE Binding Protein Bands in Boiled/Reduced and Native SSPE from Durum Wheat

**Figure 1:**
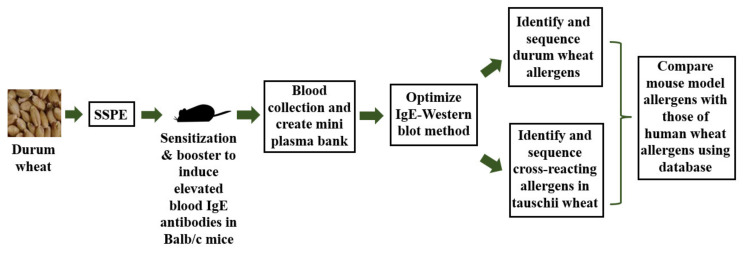
Overall experimental approach used in this study. The durum wheat flour was used to produce salt-soluble protein extract (SSPE). Groups of Balb/c mice (*n* = 20) were sensitized to SSPE using a published method. After booster injections, blood was collected at biweekly intervals. Plasma was separated and pooled to create a mini plasma bank. The anti-SSPE IgE antibody levels in the plasma were quantified by ELISA (titer: 1/2560). Then it was used to optimize an IgE-Western blot method. The IgE antibody binding protein bands present in durum wheat SSPE were identified in the Western blot. These protein bands were then sequenced by LC-MS/MS method and durum wheat allergens were identified. Using *A. tauschii* wheat SSPE, the cross-reacting allergens that bind to anti-durum wheat IgE antibodies were identified. The mouse model allergens were then compared to the human wheat allergens reported in the database.

**Figure 2:**
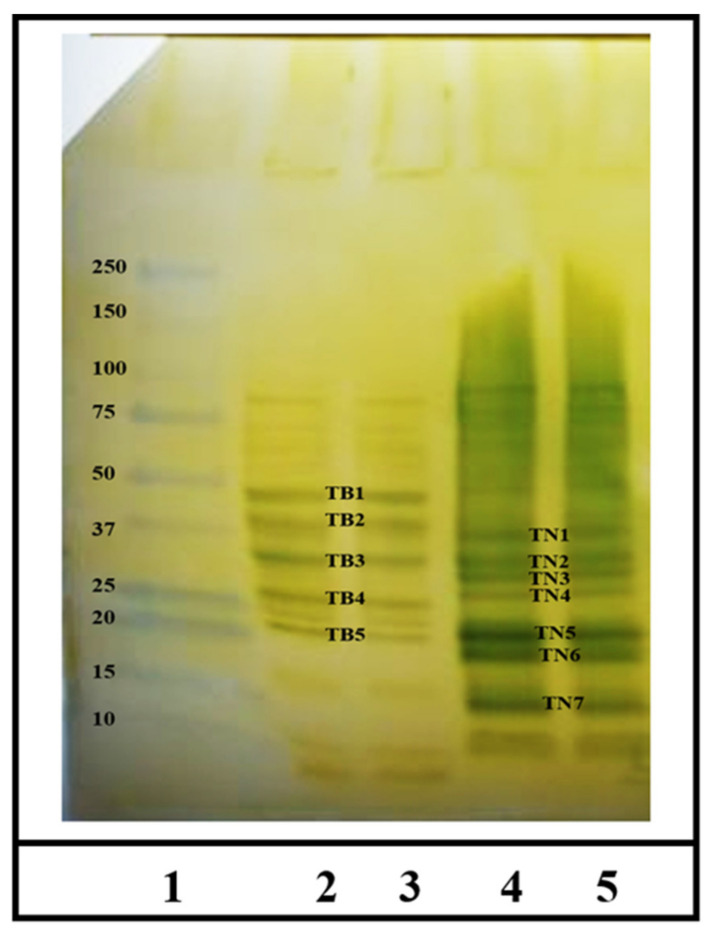
Western blot analysis of IgE binding protein bands in the salt-soluble proteins extracts from durum wheat. Figure 2 depicts data from analysis of boiled/reduced durum wheat protein extract and native durum wheat protein extract. Lane 1 = molecular weight marker (kDa); 2 and 3 = Durum wheat (AABB) boiled/reduced protein extract in duplicates. The IgE binding protein bands are labelled as Tetraploid Boiled (TB) 1 to TB5. 4 and 5 = Durum wheat (AABB) native protein extract in duplicates. The IgE binding protein bands are labelled as Tetraploid Native (TN) 1 to TN7.

The overall experimetnal approach used in this sutdy is shown in Figure 1. Specific IgE antibody levels were measured using an ultra-sensitive ELISA method we have described before [42,43]. The IgE antibody titer of the plasma was 2560. As evident, using the optimized conditions of the IgE Western blot, we found 5 distinct IgE-binding protein bands in the boiled/reduced durum SSPE (Figure 2). These were labeled Tetraploid Boiled (TB) 1 through TB5 and approximately corresponded to the following sizes, respectively: 45–48 kDa, 40–43 kDa, 30–33 kDa, 26–28 kDa, and 22–23 kDa (Figure 2). Three were 7-distinct IgE binding bands in the native durum SSPE (Figure 2) that were labelled as Tetraploid Native (TN) 1 through TN7. Their approximate sizes were, respectively: 35–37 kDa, 32–35 kDa, 28–30 kDa, 25–26 kDa, 18–20 kDa, 16–17 kDa, and 12–15 kDa (Figure 2). Notably, the last two bands were observed only in the native SSPE but not in the boiled/reduced SSPE. Native SSPE exhibited relatively stronger background activity compared to the boiled/reduced SSPE.

### 2.2. Sequencing and Identification of Allergens in Boiled/Reduced SSPE and Native SSPE from Durum Wheat

**Figure 3:**
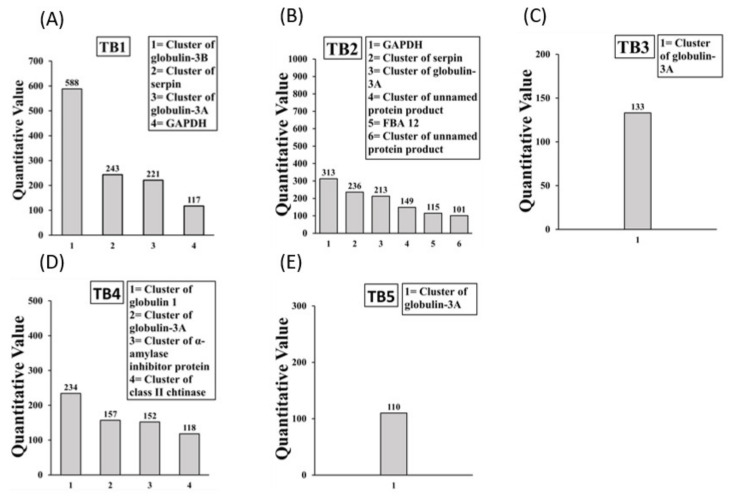
Allergens (IgE binding proteins) present in the boiled/reduced durum wheat salt-soluble protein extract. The subfigures (**A**–**E**) show the results from LC-MS/MS analysis of IgE binding protein bands TB1 to TB5 in durum wheat respectively. Specific allergens found are listed in the boxes.

Allergens present in the boiled/reduced IgE binding protein bands TB1 to TB5 are depicted in Figure 3A–E. The TB1 band contained 4 allergens. The TB2 band contained 6 allergens of which 2 were unnamed protein products. The TB3 band contained 1 allergen. The TB4 and TB5 bands contained 4 and 1 allergens, respectively. Overall, there were 9 allergens in boiled/reduced durum SSPE of which 2 were unnamed protein products, resulting in a total of 7 mouse allergens. Globulin 1, Globulin 3, and Serpin allergens appeared at multiple sizes. Detailed information on peptide sequences, position etc. is provide in Supplementary Table S1.

**Figure 4:**
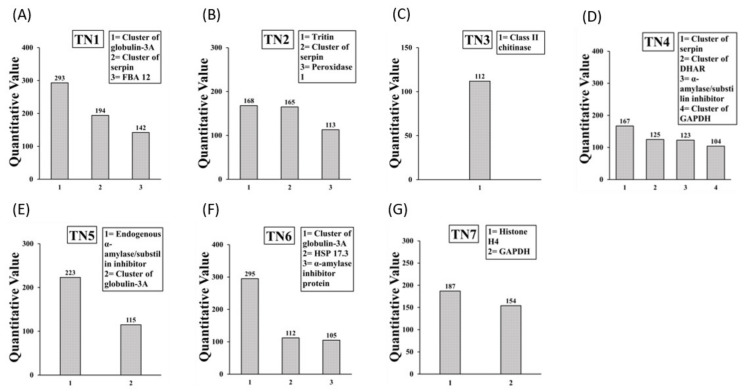
Allergens (IgE binding proteins) present in the native durum wheat salt-soluble protein extract. The subfigures (**A**–**G**) show the results from LC-MS/MS analysis of IgE binding protein bands TN1 to TN7 in durum wheat respectively. Specific allergens found are listed in the boxes.

The most abundant allergenic proteins present in the non-reduced non-boiled durum SSPE IgE binding protein bands TN1 to TN7 are shown in Figure 4A–G. The TN1 band contained 3 allergens. The TN2 band contained 3 allergens. The TR3 band contained 1 allergen. The TN4 band contained 4 allergens. The TN5 band contained 2 allergens. The TN6 band contained 3 allergens. The TN7 band contained 2 allergens. Overall, there were 12 allergens in native durum SSPE. Globulin 3A, Serpin, and GAPDH allergens appeared at multiple sizes. Notably, the following allergens present in native durum SSPE were absent in boiled/reduced durum SSPE: Tritin, Peroxidase, cluster of dehydroascorbate reductase, alpha amylase/substilin inhibitor, endogenous alpha amylase/substilin inhibitor, Histone H4, and cluster of heat shock protein 17.3. Detailed information on peptide sequences, position etc. is provide in Supplementary Table S2.

### 2.3. Identification of IgE Binding Protein Bands in Boiled/Reduced SSPE and Native SSPE from the A. tauschii Wheat

**Figure 5:**
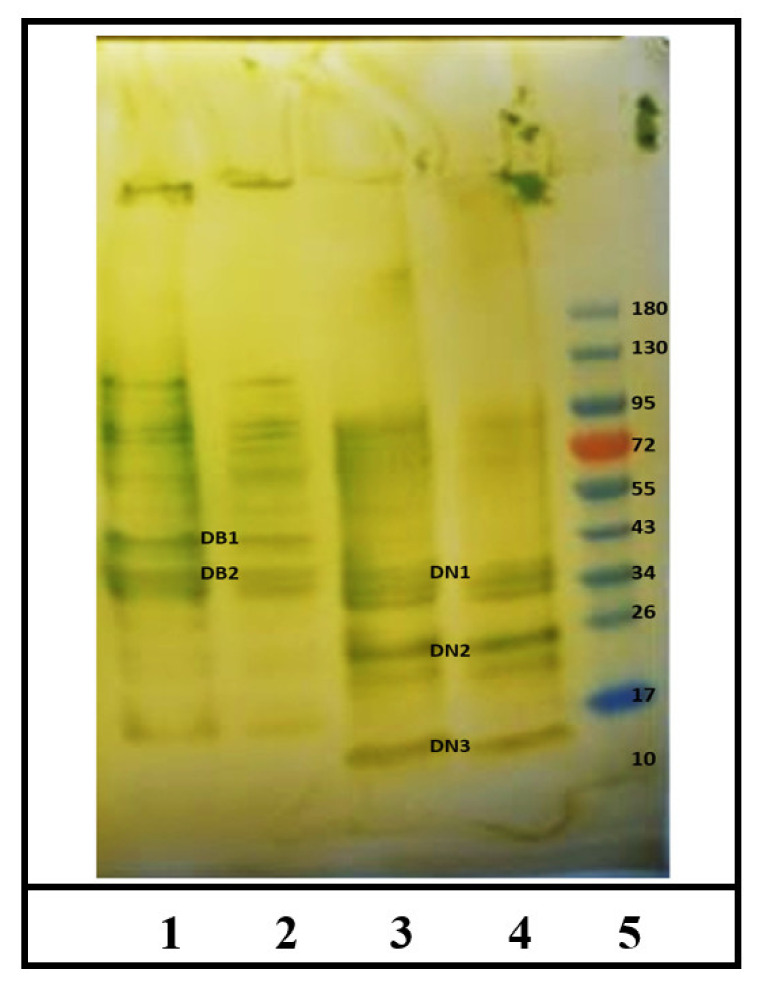
Western blot analysis of IgE binding protein bands in the salt-soluble proteins extracts from *A. tauschii* wheat. Figure 5 depicts data from analysis of boiled/reduced and native *A. tauschii* wheat protein extract. Lanes 1 & 2 = *A. tauschii* wheat (DD) boiled/reduced protein extract in duplicate. The IgE binding protein bands are labelled as Diploid Boiled (DB) 1–2. Lanes 3 & 4 = *A. tauschii* wheat (DD) native protein extract in duplicate. The IgE binding protein bands are labelled as Diploid Native (DN) 1–3. Lane 5 = molecular weight marker (kDa).

As evident, we found 2 IgE binding protein bands in the boiled/reduced *A. tauschii* SSPE (Figure 5). There were named as Diploid Boiled (DB)1 and DB2 that approximately corresponded to 40–43 kDa and 33–37 kDa, respectively. There were also few fine lines showing reactivity at the higher sizes (Figure 5). In contrast, there were 3 IgE binding bands in the native *A. tauschii* SSPE (Figure 5) that were labelled as Diploid Native (DN) 1 through DN3. Their approximate sizes were: 33–37 kDa, 24–26 kDa, and 15–16 kDa, respectively (Figure 5). Notably, DN2 and DN3 were present only in the native *A. tauschii* SSPE but not in the boiled/reduced *A. tauschii* SSPE. On the other hand, DB1 was present only in the boiled/reduced SSPE, and there was no equivalent band in the native SSPE.

### 2.4. Sequencing and Identification of Allergens in Boiled/Reduced and Native A. tauschii Wheat SSPE

**Figure 6:**
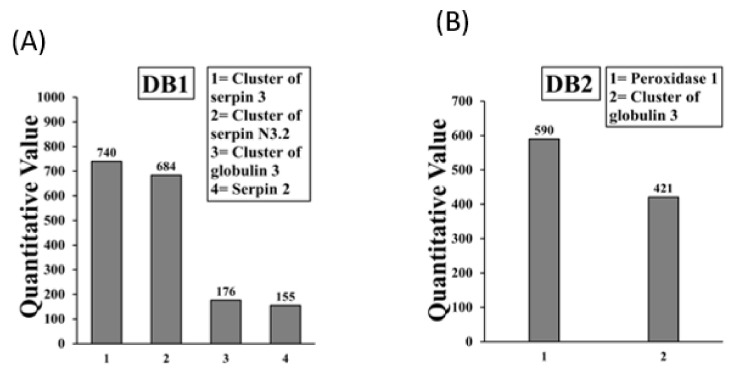
Allergens (IgE binding proteins) present in the boiled/reduced *A. tauschii* wheat salt-soluble protein extract. The subfigures (**A**,**B**) show the results from LC-MS/MS analysis of IgE binding protein bands DB1 and DB2 in *A. tauschii* wheat respectively. Specific allergens found are listed in the boxes.

Allergens present in the boiled/reduced DB1 and DB2 bands are shown in Figure 6A,B. The DB1 band contained 4 allergens (3 of which were isoforms of serpin, and Globulin 3), and the DB2 band contained 2 allergens. Overall, there were only 3 allergens (Serpins of 3 isoforms, peroxidase 1, and Globulin 3) in the boiled/reduced *A. tauschii* wheat SSPE. Detailed information on peptide sequences, position etc. is provide in Supplementary Table S3.

**Figure 7:**
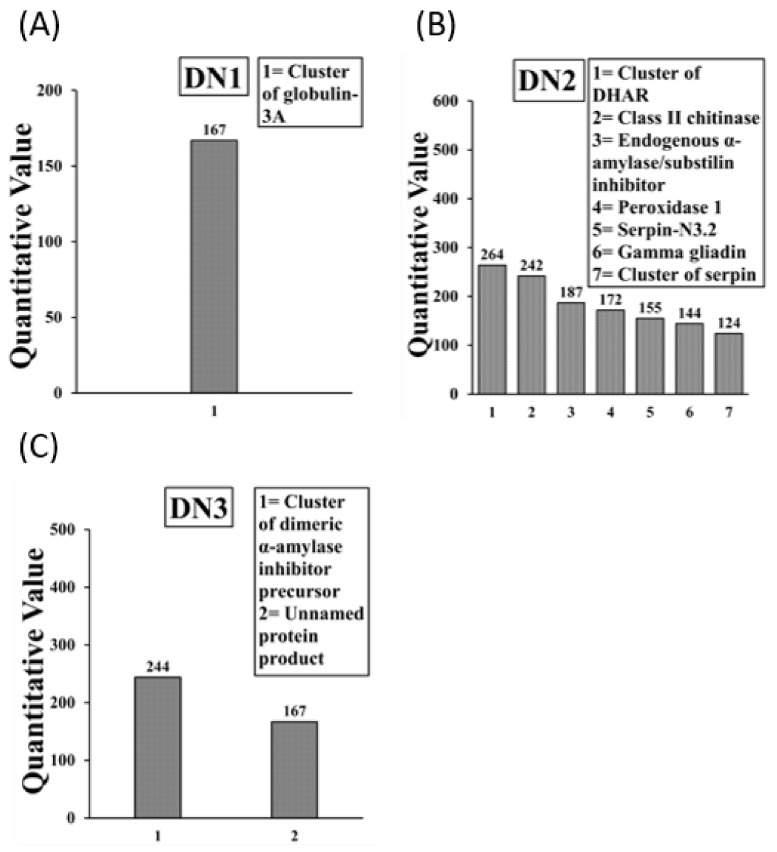
Allergens (IgE binding proteins) present in the native *A. tauschii* wheat salt-soluble protein extract. The subfigures (**A**–**C**) show the results from LC-MS/MS analysis of IgE binding protein bands DN1 to DN3 in *A. tauschii* wheat respectively. Specific allergens found are listed in the boxes.

Allergens present in the native *A. tauschii* wheat SSPE IgE-binding protein bands DN1 to DN3 are shown in Figure 7A–C. The DN1 band contained 1 allergen. The DN2 band contained 7 allergens. The DN3 band contained 2 allergens of which 1 was an unnamed product. Overall, there were 9 allergens in native *A. tauschii* wheat SSPE with one unnamed protein product resulting in 8 allergens all together. Notably, the following allergens present in native *A. tauschii* SSPE were absent in boiled/reduced *A. tauschii* SSPE: Cluster of dehydroascorbate reductase, Class II Chitinase, endogenous alpha-amylase substilin inhibitor (17–20 kDa), gamma gliadin, cluster of dimeric a-amylase inhibitor precursor, and an unnamed protein product. Detailed information on peptide sequences, position etc. is provide in Supplementary Table S4.

### 2.5. Comparison of the Wheat Allergens in the Mouse Model to Those Reported as Human Wheat Allergens in the Database

PubMed, Google Scholar and allergome.com databases were searched for evidence of the reports of human allergies to the proteins identified as allergens in this mouse model. We found that all 7 allergens identified in this mouse model in boiled/reduced durum SSPE had been reported as human allergens (Table 1). Two allergens were unnamed protein products and therefore, their relevance to human wheat allergen could not be determined. Overall, 10 out of 12 allergens found in this mouse model in the native durum SSPE had been reported as human allergens (Table 1). We found that all 3 allergens identified in the boiled/reduced *A. tauschii* SSPE had been reported as human wheat allergens (Table 2). Similarly, we found that all 8 allergens identified in this mouse model in the native *A. tauschii* SSPE had been reported as human wheat allergens (Table 2). One mouse model allergen was an unnamed protein product and therefore, its relevance to human wheat allergen could not be determined.

## 3. Discussion

Monitoring changes to wheat allergens in novel wheat varieties and wheat lines is critical to prevent inadvertent introduction of hyper-allergenic varieties via wheat breeding. However, validated methods for this purpose are unavailable at present. Towards this end, as a proof-of-concept study, here we tested the hypothesis that salt-soluble wheat allergens in our mouse model would be identical to those reported for human wheat allergy. Our data obtained using the commonly consumed durum wheat shows that mostly the same set of proteins are recognized as allergens in the mouse model as well as in humans. Furthermore, we also demonstrate that anti-durum wheat IgE antibodies also bind to similar allergens present in an ancient *A. tauschii* wheat, and those cross-reacting wheat allergens are also reported as human wheat allergens. Thus, collectively our data provides the proof-of-concept in support of our hypothesis that this mouse model may be used to monitor changes to salt-soluble wheat allergens present in the durum wheat and *A. tauschii* wheats that might be introduced by breeding.

There are seven novel findings from this study: (i) it is possible to obtain detailed information on salt-soluble wheat allergens using a mouse model of durum wheat allergy; (ii) based on susceptibility to heating, three types of allergens are present in durum wheat salt-soluble protein extract (SSPE): (a) allergens (6) that are present in the native SSPE, but not in boiled/reduced SSPE; (b) allergens (6) that are present in native SSPE, but are resistant to boiling/reducing conditions; and (c) allergens (2) that do not bind to IgE in native SSPE, but bind to IgE only in boiled/reduced SSPE (2); (iii) durum wheat-elicited IgE antibodies can identify cross-reacting allergens present in the ancient *A. tauschii* wheat; (iv) based on susceptibility to heating, similar to the durum wheat, three types of allergens are present in the *A. tauschii* wheat: (a) allergens (5) that are present in the native SSPE but are not present in boiled/reduced SSPE; (b) allergens (3) that are present in the native SSPE, but are resistant to boiling/reducing conditions; and (c) allergens (2) that do not bind to IgE in native SSPE, but bind to IgE only in boiled/reduced SSPE; (v) allergens identified in the ancient *A. tauschii* wheat are also present in the durum wheat; (vi) durum wheat contains 3 allergens (GAPDH, tritin, fructose-1,6-bisphosphate aldolase 12) that are absent in the *A. tauschii* wheat; (vi) all, but 2, durum wheat allergens, and all *A. tauschii* wheat allergens identified by us in the mouse model have been reported as human wheat allergens in the database; and (vii) two allergens (Histone H4 and cluster of heat shock protein 17.3) that are present only in the durum wheat but not in the *A. tauschii* wheat, have not been reported as human wheat allergens so far.

We used durum wheat in this study because: (i) it is a commonly consumed tetraploid wheat (AABB genome); and (ii) previously we had used durum wheat SSPE for developing and characterizing the mouse model of wheat allergy [40,42]. We also used the ancient *A. tauschii* wheat because: (i) the ancient *A. tauschii* wheat, which is a diploid wheat (DD genome), has been part of the history of wheat evolution that has resulted in today’s common bread wheat (hexaploid, AABBDD genome); and (ii) we had access to the seeds of this ancient wheat at our university repository in the wheat genetics and breeding program. Using these two types of wheats, we were able to specifically identify the allergens from these two distinct types of wheats and were able to identify the allergens present in the ancient wheat that showed IgE cross-reactivity with the durum wheat. It is remarkable that the ancient wheat contains only some of the allergens present in the durum wheat. This finding may explain our previous report where we demonstrated significantly lower IgE binding of *A. tauschii* wheat SSPE compared to the durum wheat SSPE in an IgE inhibition ELISA [44]. These in vitro data together support the idea that *A. tauschii* wheat SSPE may be relatively hypoallergenic compared to durum wheat in durum wheat-sensitized hosts. However, this remains to be tested in future in vivo studies using the mouse model.

We used the Balb/cJ mouse to generate the hyper-IgE plasma used in this study because: (i) this strain of mouse is genetically prone to develop food allergies; (ii) allergic responses to several food proteins in this mouse strain are similar to humans with food allergies to hazelnut, cashew nut, sesame, shellfish and wheat gliadins and non-gluten allergens including lipid transfer protein [28,40,45,46,47,48,49]; and (iii) we have extensively characterized the allergic response to salt-soluble wheat proteins in this model previously [40,42,44]. It is remarkable that all 7 thermal-resistant salt-soluble wheat allergens which elicit IgE responses in this model are also reported to elicit and bind to human IgE antibodies, and thus act as allergens in both species. An elegant previous study showed that purified lipid transfer protein (LTP) from wheat elicited IgE antibody response when injected into Balb/cJ mice and that subjects who were allergic to this protein showed a highly similar epitope binding structure suggesting again the similarity between the Balb/cJ mice and the human allergic responses [50]. Thus, together, previous studies along with findings from the present study further support and justify the use of the Balb/cJ mouse model for wheat allergenicity research.

The rationale for choosing salt-soluble wheat proteins in this study is as follows: wheat allergens belong to both glutens and non-gluten protein families. Salt-soluble proteins are non-glutens. Both types of allergens are important in human wheat allergic disease and therefore, both types of proteins need to be researched [22,28]. However, in this ‘proof-of-concept’ study we researched salt-soluble wheat proteins as a model wheat allergen. Using this concept, it should be also possible to study gluten allergens.

We have extensively characterized the wheat protein extracts and ensured that high quality proteins are present. We have provided the SDS-PAGE images of the two protein extracts used in this study (Supplementary Figure S1). Furthermore, we have studied their allergenicity using an IgE inhibition ELISA method and found that *A. tauschii* is indeed less allergenic [44]. This was our baseline comparison. Based on this data, we set out to identify the specific allergens using the IgE Western blot method we have described in this paper.

In the native durum SSPE we identified two mouse model wheat allergens (Histone H4, and cluster of heat shock protein) that have not been reported as human allergens. It is possible that since these 2 allergens are destroyed by boiling of SSPE, they may not be able to elicit human IgE responses since they are not expected to be present in the thermally processed wheat food products to elicit an IgE response. Since we used native SSPE to sensitize mice, these 2 proteins could elicit and bind to IgE antibodies in our study. However, it is possible for human exposures to native SSPE (and therefore to these 2 proteins) to occur via non-oral routes (eyes, airways, and skin) from non-heat-treated wheat flour at home or in the baking industries. Therefore, these two proteins may pose potential allergenicity problems in humans that is yet to be identified.

Previous studies show that depending on the type of processing, wheat allergenicity may increase or reduce or may not change [28]. For example, while boiling of wheat flour has no significant effect on allergenicity of albumins and globulins (salt-soluble wheat proteins), boiling of pasta reduced their allergenicity by about 50% as measured by human IgE Western blot analysis [23]. We also found that boiling/reducing of SSPE did not reduce allergenicity of globulins and several other salt-soluble allergens. However, we also identified several other allergens present in the native SSPE that were inactivated by boiling/reducing. In addition, we found that peroxidase present in durum wheat SSPE was inactivated by boiling/reduction; however, peroxidase present in *A. tauschii* wheat SSPE did not lose allergenicity by such processing suggesting differences in the property of the same allergen encoded by AABB vs. DD genomes. Whereas Globulin 3A (present in both durum and *A. tauschii* wheats) was resistant to boiling/reduction conditions, Globulin 1 & 3B allergens (present only in durum wheat but not in *A. tauschii* wheat) showed allergenicity only upon boiling/reducing. Interestingly, Serpins present in both durum and *A. tauschii* wheats were resistant to boiling/reducing conditions. Thus, the effects of boiling on wheat allergenicity appears to be complex. Nevertheless, our data demonstrate that the approach described here can be proactively used to identify boiling/reduction sensitive vs. resistant allergens in novel wheat varieties and wheat lines that might be created by breeding of durum and *A. tauschii* wheats [51]. Furthermore, this approach can also be used to screen existing lines and varieties to establish baselines profiles of boiling/reduction sensitive vs. resistant wheat allergens. This type of information can not only help prevent inadvertent introduction of hyper allergenic wheats but also can inform the development of novel hypo/non-allergenic wheat lines and varieties [28].

Wheat allergens belong to both non-gluten and gluten protein families. To validate a mouse model for human wheat allergenicity, it will be necessary to test both non-gluten and gluten (gliadins and glutenins) allergens in the mouse model. Here, we validate the similarity of non-gluten wheat allergens in this model to those reported for human wheat allergenicity. Previous elegant studies demonstrated that the alcohol-soluble gliadins and lipid transfer protein show allergenicity in Balb/c mice like that in human wheat allergic subjects [50,52]. However, allergenicity potential of acid-soluble glutenin in mice remains to be tested.

Currently many different wheat (sub-)species/cultivars are available on the market. There are only a few studies at present that report potential differences among them in causing human wheat allergy. Nakamura et al. (2005) studied 324 wheat varieties and reported that some are potentially hypoallergenic based on IgE binding in an ELISA [53]. Larre et al. (2011) compared salt-soluble protein allergens in diploid vs. hexaploid wheats using serum from wheat allergic subjects [54]. They reported that IgE binding was much lower for the diploid wheat. Kohno et al. 2016 developed a new wheat line lacking ω-5 gliadin locus and showed that it is less allergenic in a guinea pig model [35]. Gao et al. (2019) showed that salt-soluble protein extract from an ancient diploid wheat is significantly less allergenic than that from the tetraploid durum wheat and the hexaploid Ambassador wheat based on in vitro IgE binding [44]. Thus, there is emerging evidence that different wheats might be different in allergenicity. However, in vivo, or clinical evidence is lacking. Therefore, there is urgent need to further research this problem and carefully map the variation in clinical allergenicity among various wheat species, sub-species, and cultivars. Such research has the potential to develop hypo/non-allergenic wheat lines.

In this study, as a proof-of-concept, we used IgE enriched hyper immune plasma from durum wheat salt-soluble protein sensitized mice and studied the allergens present in durum wheat as well as cross-reacting allergens present in the ancient diploid tauschii wheat. A similar approach can be used to sensitize mice with whichever wheat variety/line one would be interested in and study the allergens using the approach we have reported here.

The mouse method described here is a novel approach to monitor changes to wheat allergens potentially caused by crossbreeding and/or genetic modification. It is not intended to replace or substitute other methods such as using human serum from wheat allergic subjects for allergen identification. However, the mouse method we describe has several advantages compared to the screening for wheat allergens using serum from wheat allergic subjects including the following: (i) in mice, exposure to wheat can be controlled completely. For example, mice can be sensitized to a particular species/sub-species/genotype (e.g., diploid vs. tetraploid vs. hexaploidy), wheat variety/lines etc. Therefore, mono sensitized IgE antibodies can be produced by this method. In contrast, such controlled exposure of humans to one wheat cultivar/species/sub-species/genotype etc., is not possible. Therefore, having mono sensitized human serum from exposure to a single type of wheat exposure is not possible to obtain from wheat allergic subjects; (ii) humans produce IgE antibodies against many grass allergens that are known to cross-react with wheat allergens [55]. Therefore, human serum containing wheat-binding IgE antibodies could be in theory from either exposure to wheat or to grass or to both. Such exposure to grass does not occur in mice where environmental and dietary exposure can be strictly controlled; (iii) the ancient diploid tauschii wheats are not commercially available. Therefore, obtaining tauschi specific IgE from humans is not possible; in this study we elicited IgE antibodies using durum wheat; however, using the same approach, mice can also be exposed to tauschii wheat in controlled conditions, and it is possible to obtain tauschii specific IgE antibodies from such mice which will not be possible to obtain from humans; and (iv) when a novel wheat cultivar/line is developed, it can also be tested in this mouse model to monitor and identify the changes to allergens. Such approach is not possible with human serum testing simply because humans would not have been exposed to the novel wheat cultivar/line at that point in time.

In this study we used our optimized IgE Western blot method to identify the allergens that bind to IgE antibodies. In most cases, for a given IgE binding band, more than one protein was identified in MS analysis. It is possible that 1 or more or all the proteins identified within band might contribute to the IgE reactivity. Therefore, the relative contribution of multiple proteins to IgE binding reactivity in Western blot remains to be determined in future studies. Therefore, the allergens identified here should be deemed as ‘*putative*’ allergens. Future studies can be conducted using single isolated proteins to determine their relative contribution to the IgE reactivity in Western blot.

There are several limitations of using a mouse model to study wheat allergy including the following: (i) human wheat allergies result from natural exposure to wheat via oral, nasal, conjunctival, or skin routes; here we sensitize mice to wheat protein using intraperitoneal injections with alum adjuvant; (ii) digestion of wheat proteins in the gut is an additional factor that influences wheat allergenicity in humans; notably, this feature is not represented in the mouse model; and (iii) some wheat-sensitized humans develop anaphylaxis after performing exercise following wheat ingestion and this is known as exercise-dependent wheat induced anaphylaxis [18]; in this mouse model to induce anaphylaxis exercise is not required; intraperitoneal injection with the wheat allergen is sufficient to elicit systemic anaphylaxis.

It is very important to validate and improve the existing mouse models of wheat allergenicity so as to simulate the allergic response as closely as possible to that in humans for a number of reasons, including the following: (i) validated mouse models of wheat allergenicity are essential to conduct pre-clinical studies testing the allergenicity of novel wheat lines, and wheat varieties developed by conventional breeding; it is important to note that validated animal models of food allergenicity have been incorporated in the decision-tree approach recommended by the FAO/WHO expert committee for establishing ‘substantial-equivalence’ of novel foods [36,37,38,39]; currently GM wheats are not commercially sold yet, but if future researchers were to consider developing GM wheats, this method would be useful [33,34,39,56,57,58]; (ii) validated animal models also have applications in the development and validation of novel hypo/non-allergenic wheat products based on food and industrial processing methods [28]; and (iii) validated mouse models will be valuable to advance the knowledge on the mechanisms of wheat allergenicity so that new methods to prevent and treat wheat allergies become available.

## 4. Materials and Methods

### 4.1. Chemicals and Reagents

The following chemicals and reagents were obtained from the sources indicated in parentheses: Biotin conjugated rat anti-mouse IgE paired antibodies and isotype standards (BD BioSciences, San Jose, CA, USA); para-Nitrophenylphosphate (Sigma, St Louis, MO, USA); streptavidin alkaline phosphatase (Jackson ImmunoResearch, West Grove, PA, USA); protein estimation reagents: bovine serum albumin standard and reagents A and B (Sigma, St Louis, MO, USA). Pre-made SDS-PAGE gels were purchased from Bio-Rad (catalog #4561094). The cellulose membranes for Western blot were purchased from Bio-Rad (catalog #1620145); as were the molecular weight markers (Bio-Rad Precision Plus Protein catalog #161-0373; Thermo Scientific PageRuler Prestained Protein Ladder Product #26616); substrate buffer (Southern Biotech TMB membrane substrate catalog #0304-01); blocking buffer (5% BSA).

### 4.2. Mice Breeding Generation of a Plant-Protein-Free Mice Colony

Balb/cJ mice (female) were generated on a plant-protein free diet (AIN-93G) and purchased from the Jackson Laboratory (Bar Harbor, ME, USA). All mice were housed in the animal facility of the Trout Food Science and Human Nutrition Building at the Michigan State University. Mice were maintained on the plant protein-free diet (AIN-93M). All mice used in this study were 4–6 weeks old at the onset of the studies. All animal procedures used were in accordance with Michigan State University policies and approved by the animal use and care review committee.

### 4.3. Preparation of Salt-Soluble Wheat Protein Extract from Durum Wheat

Durum wheat (variety Carpio) flour was used in the preparation of salt-soluble protein extract (SSPE) using the following standard method [42]. Briefly, 10 g of flour in 100 mL of 0.5 M NaCl was stirred continuously for 2 h at 20 °C followed by centrifugation (5000× *g*, 10 min) at 20 °C. The supernatant was frozen overnight at −16 °C and then freeze-dried. The protein content was quantified according to Bradford dye-binding method [59].

### 4.4. Growing Ancient A. tauschii Wheat and Preparation of Salt-Soluble Wheat Protein Extract

Using seeds stored with the Michigan State University wheat breeding program, *A. tauschii* wheat was grown at the university greenhouse. Upon harvesting, the berries were threshed, then milled. Using the wholemeal flour and following the same method described above for durum wheat, *A. tauschii* SSPE was prepared and quantified.

### 4.5. Preparing a Hyper-IgE Plasma Mini-Bank from Durum Wheat-Allergic Mice

As described before, mice (*n* = 20) were sensitized with the durum wheat SSPE to induce IgE antibody responses using the published method [44]. Briefly, animals were injected with SSPE (0.01 mg/mouse) plus alum (1 mg/mouse) four times by intraperitoneal (IP) route, on days 0, 10, 24, and 40. Specific IgE levels were measured and clinical sensitization for anaphylaxis was confirmed by IP challenge (0.5 mg/mouse) followed by rectal thermometry and determination of mucosal mast cell degranulation responses. After booster injections, blood was collected at bi-weekly intervals. Pooled plasma was prepared, and aliquots were stored at −70 °C until used in Western blot analysis. IgE levels were quantified by an optimized ELISA method as described before [44].

### 4.6. Sodium Dodecyl Sulfate Polyacrylamide Gel Electrophoresis (SDS-PAGE)

Pre-made SDS-PAGE gels (4–20%) were purchased and used in the study. The SSPE samples were analyzed under non-reduced and reduced/boiled conditions. Freeze-dried samples of durum SSPE were prepared with sample buffer (Bio-Rad catalog #161-737) and diluted with deionized water to desired concentration to produce non-reduced, non-boiled SSPE samples (hereafter referred to as “native SSPE”). Freeze-dried samples of durum SSPE were prepared with sample buffer, 5% β-mercaptoethanol, diluted with deionized water to desired concentration, and boiled at 100 °C for 10 min to produce reduced/boiled SSPE samples (hereafter referred to as “boiled SSPE”). Mini-PROTEAN TGX gels were loaded with native and entire boiled SSPE with one marker on each side of the gel and run in the running buffer at 100 volts until the dye front reached the reference line. Gels were stained using Coomassie brilliant blue dye. The method was optimized for protein quantity and run times.

### 4.7. Optimization of an IgE Western Blot Method

After SDS-PAGE the protein was transferred to a nitrocellulose membrane (Bio-Rad catalog #1620145) overnight at 4 °C. Ponceau staining was used to ensure the transfer of proteins. The membrane was washed five times using TBST, one minute each time. Blocking was done in 5% BSA for one hour at room temperature. The membrane was washed five times with TBST, one minute each. Then the membrane was incubated with the primary antibody (i.e., mouse hyper IgE plasma) in blocking buffer for three nights at 4 °C. Then the membrane was washed five times with TBST, one minute each. The membrane was then incubated with the secondary antibody (Southern Biotech Goat anti-mouse IgE-HRP catalog #1110-05) in blocking buffer for one hour. Then the membrane was washed five times with TBST, one minute each. The membrane was then incubated with the substrate solution (Southern Biotech TMB membrane substrate catalog #0304-01) for 10 min. Excess substrate was removed with TBST wash and then the signals were photographed. The method was optimized for incubation times, antibody quantities, and developing times. Plasma sample from non-allergic control mice was used as the negative control.

The pooled hyper IgE plasma was used in optimizing the IgE Western blot method. In order ensure that the durum wheat extract used in this study indeed contain human IgE reacting epitopes, we have tested and confirmed the IgE reactivity of the extracts used in this study using plasma from wheat allergic subject. Durum wheat SSPE was used with and without boiling/reducing and subjected to SDS-PAGE analysis. The following conditions were carefully optimized: (1) hyper IgE plasma dilution to use in the Western blot; the optimized amount of 35 ul of the hyper IgE plasma per 10 mL of blocking buffer was found to give a clear signal with minimal background activity; (2) incubation period for the primary antibody was tested at 1 day vs. 3 days duration and the latter was found to yield the best outcome; and (3) amount of secondary antibody was optimized using different amounts, and 40 μL per 10 mL blocking buffer was found to yield the signals.

### 4.8. Identification of Wheat Allergens by LC-MS/MS Sequencing Method

Protein bands corresponding to IgE binding bands in the Western blot were removed using sterile scalpel blades. A separate scalpel blade was used for each band to prevent cross-contamination. Protein bands were stored in 100 μL of 5% acetic acid until used in LC-MS/MS method at the Michigan State University Proteomics Core Facility as follows.

Gel bands were digested according to Shevchenko et al. (1996) with modifications [60]. Briefly, gel bands were dehydrated using 100% acetonitrile and incubated with 10 mM dithiothreitol in 100 mM ammonium bicarbonate at a pH of ~8 at 56 °C for 45 min. The bands were then dehydrated again and incubated in darkness with 50 mM iodoacetamide in 100 mM ammonium bicarbonate for 20 min. Gel bands were then washed with ammonium bicarbonate and dehydrated again. Sequencing grade modified trypsin was prepared to 0.01 μg/μL in 50 mM ammonium bicarbonate and ~50 μL of this was added to each gel band, completely submerging the band. Bands were then incubated at 37 °C overnight. Peptides were extracted from the gel via water bath sonication in a solution of 60% ACN/1% TCA and vacuum dried to 2 μL. Peptides were then re-suspended in 2% acetonitrile/1% TFA to 20 μL. From this, 5 uL were injected by a Thermo EASYnLC 1000 onto a Thermo Acclaim PepMap RSLC 0.075 mm × 250 mm C18 column and eluted over 35 min with a gradient of 6% B to 32% B in 24 min, ramping to 90% B at 35 min and held at 90% B for the duration of the run at a constant flow rate of 300 nL/min. (Buffer A = 99.9% Water/0.1% Formic Acid, Buffer B = 99.9% Acetonitrile/0.1% Formic Acid). Column temperature was maintained at a constant 50 o C using an integrated column heater (PRSO-V1, Sonation GmbH, Biberach, Germany).

Eluted peptides were sprayed into a ThermoFisher Q-Exactive mass spectrometer using a FlexSpray ion source. Survey scans were taken in the Orbi trap (70000 resolution, determined at *m*/*z* 200) and the top ten ions in each survey scan were then subjected to automatic higher energy collision induced dissociation (HCD) with fragment spectra acquired at a resolution of 17,500. The resulting MS/MS spectra were converted to peak lists using Mascot Distiller, v2.7 and searched against a database containing protein sequences from *T. aestivum* appended with common laboratory contaminants. The Mascot output was analysed using Scaffold v. 4.8.8 to probabilistically validate protein identifications. Assignments validated using the Scaffold 1% FDR confidence filter were considered true. Most abundant proteins were identified with a quantitative value of ≥100.

### 4.9. Comparison of Mouse Wheat Allergens to Human Wheat Allergens Reported in the Literature

Each protein identified as an allergen in the mouse model was checked in the allergome.com, Google Scholar, and PubMed databases. If it was found to be a reported as an allergen in humans, then the information was recorded as a ‘reported human wheat allergen’. If it was not listed in any of these databases, then it was deemed to be ‘not-reported’ as a human wheat allergen.

## 5. Conclusions

Here we demonstrate that all but two salt-soluble wheat allergens in durum wheat and all salt-soluble IgE cross-reacting allergens in an ancient *A. tauschii* wheat, all identified through a wheat-allergic mouse model, are indeed identical to those reported as allergens in human wheat-allergic subjects. This study also further supports the use of the Balb/cJ mouse model to advance scientific knowledge on wheat allergenicity and to use it as a pre-clinical testing tool to assess and monitor changes to wheat allergens occurring either naturally by random mutations or by human intervention such as breeding of new wheat varieties. This method will be a useful tool to assess the effects of food processing on wheat allergenicity, advance mechanisms of wheat allergenicity, and develop novel methods to prevent/treat wheat allergies.

## Figures and Tables

**Table 1:** Durum wheat allergens identified in the mouse model vs. reported wheat allergens in humans.

Durum Mouse Allergens	Accession Number	Human Allergen
***Boiled/reduced SSPE (7)***		
Tri Glo (Globulin 3 A, B)	AFM30909.1 [3], ACJ65515.1 [5]	Yes
Cluster of Globulin 1 *,**	ABG68034.1 [2]	Yes
Tri a 33 (Serpin)	CAB52710.1 [5], CAA90071.1 [5]	Yes
Tri a 34 (GAPDH)	ANW11922.1 (+1), ALE18232.1 [3]	Yes
Tri a aASI (Endogenous α-amylase/substilin inhibitor)	AAR10959.1 [4], IAAS_WHEAT (+1)	Yes
Tri a Chitinase	AAX83262.1 [13]	Yes
Cluster of fructose-1,6-bisphophate aldolase 12 **	AVL25144.1 [5]	Yes
***Native SSPE (12)***		
Tri Glo (Globulin 3 A)	AFM30909.1 [4]	Yes
Tri a 33 (Serpin)	CAB52710.1 [7], CAA90071.1 [6]	Yes
Tri a Tritin	BAA0248.1	Yes
Tri a Peroxidase 1	AAM88383.1 (+4)	Yes
Tri a Chitinase	AAX83262.1	Yes
Cluster of dehydroascorbate reductase **	AAL71851.1 [2]	Yes
Tri a aASI (Endogenous α-amylase/substilin inhibitor)	AAR10959.1 (+2)	Yes
Tri a aASI (Endogenous α-amylase/substilin inhibitor)	P16347.1 (+1)	Yes
Cluster of GAPDH **	ALE18233.1 [3], ANW11922.1 (+1)	Yes
Histone H4 **	AAA34292.1 (+21)	?
Cluster of fructose-1,6-bisphophate aldolase 12 **	AVL25144.1 [5]	Yes
Cluster of heat shock protein 17.3 **	CAA41218.1 [5]	?

SSPE: Salt-soluble protein extract; * These proteins showed IgE binding only in boiled/reduced SSPE but not in native SSPE; underlined allergens were present only in the native SSPE; ** For this protein technical allergen name (Tria a #) is not available at present.

**Table 2:** Ancient tauschii wheat allergens identified in the mouse model vs. reported wheat allergens in humans.

*A. tauschii* Mouse Allergens	Accession Number	Human Allergen
***Boiled/reduced SSPE (3)***		
Tri a 33 (Serpin 2 *, 3 *, N3.2)	ACN59484.1 (+1), ACN59485.1 [6], AFC89429.1 [5]	Yes
Tri Glo (Globulin 3)	ACJ65514.1 [4]	Yes
Tri a Peroxidase 1	AAM88383.1 (+4)	Yes
***Raw SSPE (8)***		
Tri Glo (Globulin 3A)	AFM30909.1 [4]	Yes
Cluster of dehydroascorbate reductase **	ACV89491.1 [2]	Yes
Tri a Chitinase	AAX83262.1	Yes
Tri a aASI (Endogenous α-amylase/substilin inhibitor)	P16347.1 (+1)	Yes
Tri a Peroxidase 1	AAM8838.1 (+4)	Yes
Tri a 33 (Serpin N3.2)	AFC89429.1	Yes
Tri a 20 (Gamma gliadin)	ABO37959.1	Yes
Tri a 28 (dimeric α-amylase inhibitor precursor)	ABF93411.1 [8]	Yes

SSPE: Salt-soluble protein extract; * These proteins showed IgE binding only in boiled/reduced SSPE but not in native SSPE; underlined allergens were present only in the native SSPE; ** For this protein technical allergen name (Tria a #) is not available at present.

## Data Availability

Data has been provided in the paper as well as in the Supplementary Tables and Figure.

## Supplementary Materials

The following supporting information can be downloaded at: https://www.mdpi.com/article/10.3390/ijms23126505/s1.
