# Supplementary material for: A Mouse-Based Method to Monitor Wheat Allergens in Novel Wheat Lines and Varieties Created by Crossbreeding: Proof-of-Concept Using Durum and A. tauschii Wheats
Source: Int J Mol Sci. 2022 Jun 10;23(12):6505. doi: 10.3390/ijms23126505 (PMC9224339; doi:10.3390/ijms23126505)
Supplement: Supplementary file 1 [file ijms-23-06505-s001.zip › Supl_TableS1_TB1-TB5.pdf]

Table S1. Peptides sequences, positions, and coverage for wheat allergens identified in boiled/reduced tetraploid durum wheat.

IgE-binding band	Protein	Sequence	Position	Percent coverage
TB1	Globulin-3B [Triticum aestivum]	AKDQQDEGFVAGPEQQEQER	459-478	48.60%
		AKDQQDEGFVAGPEQQEQERGDR	459-481	48.60%
		ALRPFDEVSR	147-156	48.60%
		ASEEQLR	265-271	48.60%
		DQQDEGFVAGPEQQEQER	461-478	48.60%
		DQQDEGFVAGPEQQEQERGDR	461-481	48.60%
		EASEGGQGHHWPLPPFR	276-292	48.60%
		EDQQQHGRHEQEEQGHSHGR	41-60	48.60%
		EEHGRHEQGR	99-108	48.60%
		EVQEVFR	452-458	48.60%
		GEGERDEEHGDSR	112-124	48.60%
		GRHGEGEREEEEGR	75-88	48.60%
		GRHGEGEREEHGR	91-103	48.60%
		HEQEEQGHSHGR	49-60	48.60%
		HGEGEREEEEGR	77-88	48.60%
		HGEGEREEEEGRGR	77-90	48.60%
		HGEGEREEHGR	93-103	48.60%
		HGEGEREEHGRHEQGR	93-108	48.60%
		HGEGGREEEQGR	61-72	48.60%
		LYEADAR	315-321	48.60%
		PLLASLSK	222-229	48.60%
		PLLASLSKR	222-230	48.60%
		PYVFGPR	126-132	48.60%
		QGQEEEESISIVR	252-264	48.60%
		RGEGERDEEHGDSR	111-124	48.60%
		RPFDEVSR	149-156	48.60%
		RPYVFGPR	125-132	48.60%
		RQGQEEEESISIVR	251-264	48.60%
		RSYSVR	208-213	48.60%
		SDHGFVK	140-146	48.60%

		SFHALAQHDVR	322-332	48.60%
		TSDEQLDR	238-245	48.60%
TB1	Globulin-3B [Triticum aestivum]	VAIMEVNPR	166-174	48.60%
		VAVANITPGSMTAPYLNTQSFK	333-354	48.60%
TB1	Serpin [Triticum aestivum]	AAEVTAQVNSWVEK	138-151	60.30%
		AEAQSVDFQTK	127-137	60.30%
		ATTLATDVR	2-10	60.30%
		DILPAGSISNTTR	159-171	60.30%
		DQLVATLGEGEAER	62-75	60.30%
		EDTSGVVLFIGHVVNPLR	379-396	60.30%
		GAWTDQFDSR	182-191	60.30%
		HLGLQLPFSDEADLSEMVDSPMPQGLR	301-327	60.30%
		ISFGIEASDLLK	289-300	60.30%
		ISSVFHK	328-334	60.30%
		LHALAEQVVQFVLADASYADSPR	76-98	60.30%
		LSAEPELLER	261-270	60.30%
		LSIAHQTR	11-18	60.30%
		LVLGNALYFK	172-181	60.30%
		QYISSSDGLK	217-226	60.30%
		SGIWSLAEK	252-260	60.30%
		SIAHQTR	12-18	60.30%
		TFVEVNETGTEAAAATIAK	335-353	60.30%
		VLKLPYK	227-233	60.30%
		VTTGLIK	152-158	60.30%
		VTTGLIKDILPAGSISNTTR	152-171	60.30%
		YKAEAQSVDFQTK	125-137	60.30%
		YKAEAQSVDFQTKAAEVTAQVNSWVEK	125-151	60.30%
		AAEVTTQVNSWVEK	138-151	50.00%
		AETQSVDFQTK	127-137	50.00%
		ATTLATDVR	2-10	50.00%
		DILPPGSIDNTTK	159-171	50.00%
		DQLVATLGTGK	62-72	50.00%
		EDISGVVLFMGHVVNPLLSS	379-398	50.00%
		GAWTEQFDSYGTK	182-194	50.00%

		IKDILPPGSIDNTTK	157-171	50.00%
TB1	Serpin [Triticum aestivum]	ISFGIEASDLLK	289-300	50.00%
		LASTISSNPK	23-32	50.00%
		LSAEPDFLER	261-270	50.00%
		LSIAHQTR	11-18	50.00%
		LVLANALYFK	172-181	50.00%
		QFFMYILLPEAPGGLSSLAEK	240-260	50.00%
		SIAHQTR	12-18	50.00%
		VLKLPYK	227-233	50.00%
		VSSVFHQAFVEVNEQGTEAAASTAIK	328-353	50.00%
		YKAETQSVDFQTK	125-137	50.00%
TB1	Globulin-3A [Triticum aestivum]	AFVVPGLTDADGVGYVAQGEGVLTVIENGEK	182-212	71.40%
		AKDQQDEGFVAGPEQQQEHER	542-562	71.40%
		AKDQQDEGFVAGPEQQQEHERGDR	542-565	71.40%
		ALRPFDEVSR	154-163	71.40%
		ASEEQLR	307-313	71.40%
		CQQDRPR	48-54	71.40%
		DDQQQHGR	66-73	71.40%
		DDQQQHGRHEQEEQGR	66-81	71.40%
		DQQDEGFVAGPEQQQEHER	544-562	71.40%
		DQQDEGFVAGPEQQQEHERGDR	544-565	71.40%
		DTFNLLEQR	339-347	71.40%
		DTFNLLEQRPK	339-349	71.40%
		EVQEVFR	535-541	71.40%
		FQYFSAK	257-263	71.40%
		FQYFSAKPL	257-265	71.40%
		FQYFSAKPLLASLSK	257-271	71.40%
		GDSRDTFNLLEQRPK	335-349	71.40%
		GEGERDEEHGDGR	119-131	71.40%
		GQGEREEEQGR	103-113	71.40%
		GSAFVVPPGHPVVEIASSR	470-488	71.40%
		GSGSESEEEQDQQR	446-459	71.40%
		GSGSESEEEQDQQRYETVR	446-464	71.40%
		GSSNLQVVCFEINAER	489-504	71.40%

TB1	Globulin-3A [Triticum aestivum]	GSSNLQVVCFEINAERNER	489-507	71.40%
		HEQEEQGR	74-81	71.40%
		HEQEEQGRGHGR	74-85	71.40%
		HGEGEREEEQGR	86-97	71.40%
		ILHTISVPGK	247-256	71.40%
		LDDPAQELAFGR	520-531	71.40%
		LDDPAQELAFGRPAR	520-534	71.40%
		LGSLLGSR	285-292	71.40%
		LYEADAR	357-363	71.40%
		PLLASLSK	264-271	71.40%
		PLLASLSKR	264-272	71.40%
		PYVFGPR	133-139	71.40%
		QASEGDQGHHWPLPPFR	318-334	71.40%
		QGDVIVAPAGSIMHLANTDGR	219-239	71.40%
		QGDVIVAPAGSIMHLANTDGRR	219-240	71.40%
		QGKEEEEK	293-300	71.40%
		REQEHGK	421-427	71.40%
		REQEHGKGR	421-429	71.40%
		RGEGERDEEHGDGR	118-131	71.40%
		RGQGEREEEQGR	102-113	71.40%
		RGSGSESEEEQDQQR	445-459	71.40%
		RGSGSESEEEQDQQRYETVR	445-464	71.40%
		RKLVIAK	240-246	71.40%
		RPFDEVSR	156-163	71.40%
		RPYVFGPR	132-139	71.40%
		RRGSGSESEEEQDQQR	444-459	71.40%
		RSYTVR	213-218	71.40%
		RVLTAALK	272-279	71.40%
		SDHGFVK	147-153	71.40%
		SEEEEDDRR	432-440	71.40%
		SEEEEDDRRQQR	432-443	71.40%
		SFHALAQHDVR	364-374	71.40%
		VAIMEVNPR	173-181	71.40%
	Globulin-3A	VAVANITPGSMTAPYLNTQSFK	375-396	71.40%

TB1	[Triticum aestivum]	VLTAALK	273-279	71.40%
		WRSEEEEDDRR	430-440	71.40%
TB1	Glyceraldehyde-3-phosphate dehydrogenase [Triticum aestivum]	AAIKEESEGNLK	262-273	81.00%
		AASFNIIPSSTGAAK	203-217	81.00%
		AGIALNDNFVK	301-311	81.00%
		DAPMFVCGVNEK	129-140	81.00%
		DKAAAHIK	108-115	81.00%
		EESEGNLK	266-273	81.00%
		EVAVFGCR	75-82	81.00%
		EYKSDIDIVSNASCTTNCLAPLAK	141-164	81.00%
		FGIVEGLMTTVHAMTATQK	170-188	81.00%
		GILGYVDEDLVSTDFQGDNR	274-293	81.00%
		IGINGFGR	6-13	81.00%
		KVIISAPSK	120-128	81.00%
		LAKPATYDQIK	251-261	81.00%
		LTGMAFR	230-236	81.00%
		LVSWYDNEWGYSTR	312-325	81.00%
		NPEEIPWAAAGAEYVVESTGVFTDKDK	83-109	81.00%
		PATYDQIK	254-261	81.00%
		SDIDIVSNASCTTNCLAPLAK	144-164	81.00%
		SSIFDAK	294-300	81.00%
		TLLFGEK	68-74	81.00%
		TLLFGEKEVAVFGCR	68-82	81.00%
		TVDGPSSK	189-196	81.00%
		VALQSPDVELVAVNDPFITTDYMTYMFK	21-48	81.00%
		VIISAPSK	121-128	81.00%
		VLPELNGK	222-229	81.00%
		VPTVDVSVVDLTVR	237-250	81.00%
		VVDLIR	326-331	81.00%
		YDTVHGQWK	49-57	81.00%
TB1	Glyceraldehyde-3-phosphate dehydrogenase [Triticum aestivum]	AASFNIIPSSTGAAK	203-217	65.30%
		AASYDDIKK	254-262	65.30%
		AGIALNDHFVK	301-311	65.30%
		DDKTLLFGEKPVTVFGVR	65-82	65.30%

		DKAAAHLK	108-115	65.30%
		GIMGYVEEDLVSTDFVGDSR	274-293	65.30%
		IGINGFGR	6-13	65.30%
		IINDNFGIIEGLMTTVHAITATQK	165-188	65.30%
		KVVISAPSK	120-128	65.30%
		LTGMSFR	230-236	65.30%
		LVSWYDNEWGYSNR	312-325	65.30%
		NPEEIPWGEAGADYVVESTGVFTDKDK	83-109	65.30%
		SSIFDAK	294-300	65.30%
		TLLFGEK	68-74	65.30%
		TLLFGEKPVTVFGVR	68-82	65.30%
		TVDGPSSK	189-196	65.30%
		VLPELNGK	222-229	65.30%
		VPTVDVSVVDLTVR	237-250	65.30%
		VVDLIR	326-331	65.30%
		VVISAPSK	121-128	65.30%
		YDTVHGHWK	49-57	65.30%
TB2	Glyceraldehyde-3-phosphate dehydrogenase, partial [Triticum aestivum]	AASFNIIPSSTGAAK	202-216	76.10%
		AASFNIIPSSTGAAKAVGK	202-220	76.10%
		AASYDDIK	253-260	76.10%
		AGIALNDHFVK	300-310	76.10%
		DAPMFVVGVNEDK	128-140	76.10%
		DAPMFVVGVNEDKYTSDVNIVSNASCTTNCLAPLAK	128-163	76.10%
		DDKTLLFGEKPVTVFGVR	64-81	76.10%
		GIMGYVEEDLVSTDFVGDSR	273-292	76.10%
		IGINGFGR	5-12	76.10%
		IINDNFGIIEGLMTTVHAITATQK	164-187	76.10%
		KVVISAPSK	119-127	76.10%
TB2	Glyceraldehyde-3-phosphate dehydrogenase [Triticum aestivum]	LKGIMGYVEEDLVSTDFVGDSR	271-292	76.10%
		LTGMSFR	229-235	76.10%
		LVSWYDNEWGYSNR	311-324	76.10%
		NPEEIPWGEAGADYVVESTGVFTDKDK	82-108	76.10%
		PVTVFGVR	74-81	76.10%
		SSIFDAK	293-299	76.10%

		TLLFGEK	67-73	76.10%
		TLLFGEKPVTVFGVR	67-81	76.10%
		TTNCLAPLAK	154-163	76.10%
		TVDGPSSK	188-195	76.10%
		VLPELNGK	221-228	76.10%
		VLPELNGKLTGMSFR	221-235	76.10%
		VPTVDVSVVDLTVR	236-249	76.10%
		VVDLIR	325-330	76.10%
		VVISAPSK	120-127	76.10%
		YDTVHGHWK	48-56	76.10%
		AAIKEESEGNLK	262-273	76.60%
		AASFNIIPSSTGAAK	203-217	76.60%
		AASFNIIPSSTGAAKAVGK	203-221	76.60%
		AGIALNDNFVK	301-311	76.60%
		DAPMFVCGVNEK	129-140	76.60%
		DAPMFVCGVNEKEYK	129-143	76.60%
		EVAVFGCR	75-82	76.60%
		EYKSDIDIVSNASCTTNCLAPLAK	141-164	76.60%
		FGIVEGLMTTVHAMTATQK	170-188	76.60%
		GILGYVDEDLVSTDFQGDNR	274-293	76.60%
		HMHSSK	332-337	76.60%
		KVIISAPSK	120-128	76.60%
		KVIISAPSKDAPMFVCGVNEK	120-140	76.60%
		LAKPATYDQIK	251-261	76.60%
		LTGMAFR	230-236	76.60%
		LVSWYDNEWGYSTR	312-325	76.60%
		NPEEIPWAAAGAEYVVESTGVFTDKDK	83-109	76.60%
TB2	Glyceraldehyde-3-phosphate dehydrogenase [Triticum aestivum]	PATYDQIK	254-261	76.60%
		SDIDIVSNASCTTNCLAPLAK	144-164	76.60%
		SSIFDAK	294-300	76.60%
		TLLFGQK	68-74	76.60%
		TTNCLAPLAK	155-164	76.60%
		TVDGPSSK	189-196	76.60%
		VIISAPSK	121-128	76.60%

		VLPELNGK	222-229	76.60%
		VLPELNGKLTGMAFR	222-236	76.60%
		VPTVDVSVVDLTVR	237-250	76.60%
		VVDLIR	326-331	76.60%
		YDTVHGQWK	49-57	76.60%
TB2	Serpin [Triticum aestivum]	AAEVATQVNSWVEK	138-151	44.50%
		ATTLATDVR	2-10	44.50%
		GAWTDQFDSSGTK	182-194	44.50%
		IKDILPSGSVDNTTK	157-171	44.50%
		ISFETEASDLLK	289-300	44.50%
		LASTISSNPK	23-32	44.50%
		LSAEPDFLER	261-270	44.50%
		LSAEPDFLERHIPR	261-274	44.50%
		LVLANALYFK	172-181	44.50%
		PSFQELAVCK	115-124	44.50%
		QFKLPK	281-286	44.50%
		QFSMYILLPEAPGGLSNLAEK	240-260	44.50%
		QYLSSSDGLK	217-226	44.50%
		VLKLPYK	227-233	44.50%
		VSSVFHQAFVEVNEQGTEAAASTAIK	328-353	44.50%
		AAEVTAQVNSWVEK	138-151	62.30%
		AEAQSVDFQTK	127-137	62.30%
		ATTLATDVR	2-10	62.30%
		DILPAGSISNTTR	159-171	62.30%
		DQLVATLGEGEAER	62-75	62.30%
		EDTSGVVLFIGHVVNPLR	379-396	62.30%
TB2	Serpin [Triticum aestivum]	GAWTDQFDSR	182-191	62.30%
		HLGLQLPFSDEADLSEMVDSPMPQGLR	301-327	62.30%
		ISFGIEASDLLK	289-300	62.30%
		ISSVFHK	328-334	62.30%
		LHALAEQVVQFVLADASYADSPR	76-98	62.30%
		LSAEPELLER	261-270	62.30%
		LVLGNALYFK	172-181	62.30%
		PSFQELAVCK	115-124	62.30%

		QFKLPK	281-286	62.30%
		QYISSSDGLK	217-226	62.30%
		SGIWSLAEK	252-260	62.30%
		TFVEVNETGTEAAAATIAK	335-353	62.30%
		VLKLPYK	227-233	62.30%
		VTTGLIK	152-158	62.30%
		VTTGLIKDILPAGSISNTTR	152-171	62.30%
		YKAEAQSVDFQTK	125-137	62.30%
		YKAEAQSVDFQTKAAEVTAQVNSWVEK	125-151	62.30%
TB2	Globulin-3A [Triticum aestivum]	AFVVPGLTDADGVGYVAQGEGVLTVIENGEK	182-212	58.20%
		AKDQQDEGFVAGPEQQQEHER	542-562	58.20%
		AKDQQDEGFVAGPEQQQEHERGDR	542-565	58.20%
		ALRPFDEVSR	154-163	58.20%
		CVQECRDDQQQHGR	60-73	58.20%
		DDQQQHGR	66-73	58.20%
		DTFNLLEQRPK	339-349	58.20%
		EQEHGK	422-427	58.20%
		EVQEVFR	535-541	58.20%
		FQYFSAK	257-263	58.20%
		FQYFSAKPLLASLSK	257-271	58.20%
		GDSRDTFNLLEQRPK	335-349	58.20%
		GSAFVVPPGHPVVEIASSR	470-488	58.20%
		GSGSESEEEQDQQRYETVR	446-464	58.20%
		GSSNLQVVCFEINAER	489-504	58.20%
		HGEGEREEEQGR	86-97	58.20%
TB2	Globulin-3A [Triticum aestivum]	ILHTISVPGK	247-256	58.20%
		LAVVLEGEGEVEIVCPHLGR	397-416	58.20%
		LDDPAQELAFGR	520-531	58.20%
		LDDPAQELAFGRPAR	520-534	58.20%
		LGSLLGSR	285-292	58.20%
		PLLASLSK	264-271	58.20%
		PLLASLSKR	264-272	58.20%
		PYVFGPR	133-139	58.20%
		QASEGDQGHHWPLPPFR	318-334	58.20%

		QGDVIVAPAGSIMHLANTDGR	219-239	58.20%
		QGDVIVAPAGSIMHLANTDGRR	219-240	58.20%
		QGKEEEEK	293-300	58.20%
		REQEHGK	421-427	58.20%
		RGSGSESEEEQDQQR	445-459	58.20%
		RPYVFGPR	132-139	58.20%
		RQASEGDQGHHWPLPPFR	317-334	58.20%
		RVLTAALK	272-279	58.20%
		SEEEEDDRR	432-440	58.20%
		SFHALAQHDVR	364-374	58.20%
		VAIMEVNPR	173-181	58.20%
		VLTAALK	273-279	58.20%
		VWLAGR	508-513	58.20%
TB2	Unnamed protein product [Triticum aestivum]	ADLNVPLDDAQK	24-35	76.60%
		AHASTEGVTK	156-165	76.60%
		ASIPTIK	44-50	76.60%
		ELDYLVGAVANPK	179-191	76.60%
		FAAGTDAIAK	332-341	76.60%
		FAAIVGGSK	194-202	76.60%
		FLRPSVAGFLMQK	166-178	76.60%
		FYKEEEKNDPEFAK	123-136	76.60%
		GVTTIIGGGDSVAAVEK	350-366	76.60%
		IGVIESLLAK	207-216	76.60%
		ITDDTR	36-41	76.60%
TB2	Unnamed protein product [Triticum aestivum]	IVPATAIPDGWMGLDVGPDSIK	285-306	76.60%
		KLASVADLYVNDAFGTAHR	137-155	76.60%
		KPFAAIVGGSK	192-202	76.60%
		LAAALPDGGVLLLENVR	106-122	76.60%
		LASVADLYVNDAFGTAHR	138-155	76.60%
		LLLPTDVVVADK	265-276	76.60%
		LLLPTDVVVADKFAADAESK	265-284	76.60%
		MSHISTGGGASLELLEGKPLPGVLALDEA	373-401	76.60%
		NDPEFAKK	130-137	76.60%
		QLAELTGK	342-349	76.60%

		RSVGTLGEADLK	5-16	76.60%
		SLVEEDKLELATSLIETAK	241-259	76.60%
		SVGTLGEADLK	6-16	76.60%
		SVGTLGEADLKGK	6-18	76.60%
		TFAEALDTTK	307-316	76.60%
		TVIWNGPMGVFEFEK	317-331	76.60%
		VILASHLGR	59-67	76.60%
		VILASHLGRPK	59-69	76.60%
TB2	Fructose-1,6-bisphosphate aldolase 12 [Triticum aestivum]	AGNVLPGIK	95-103	62.60%
		AGNVLPGIKVDK	95-106	62.60%
		ALNDQHVLLEGTLLKPNMVTPGSDSK	211-236	62.60%
		ALNDQHVLLEGTLLKPNMVTPGSDSKK	211-237	62.60%
		ANSEATLGTYK	328-338	62.60%
		FASINVENVEDNR	40-52	62.60%
		FASINVENVEDNRR	40-53	62.60%
		GDATLAEGASESLHVK	339-354	62.60%
		GGKPFVDILK	85-94	62.60%
		GILAADESTGTIGK	25-38	62.60%
		GILAADESTGTIGKR	25-39	62.60%
		GTIELAGTNGETTTQGFDDLGK	107-128	62.60%
		GTIELAGTNGETTTQGFDDLGKR	107-129	62.60%
		IGATEPSQLSIDQNAQGLAR	149-168	62.60%
		KPWNLSFSFGR	288-298	62.60%
TB2	Fructose-1,6-bisphosphate aldolase 12 [Triticum aestivum]	KVAPEVIAEYTVR	237-249	62.60%
		NAAYIGTPGK	15-24	62.60%
		PFVDILK	88-94	62.60%
		RFASINVENVEDNRR	39-53	62.60%
		TENEEK	312-317	62.60%
		TVPAAVPAIVFLSGGQSEEEATLNLNAMNK	254-283	62.60%
		VAPEVIAEYTVR	238-249	62.60%
		VDKGTIELAGTNGETTTQGFDDLGK	104-128	62.60%
		VDKGTIELAGTNGETTTQGFDDLGKR	104-129	62.60%
		YKDELIK	8-14	62.60%
		AEQMLIHNESR	104-114	60.60%

TB2	Unnamed protein product [Triticum aestivum]	ALLPFFDSAYQGFASGSLDKDAQSVR	258-283	60.60%
		DSAMFDEWTVELK	354-366	60.60%
		EQLFDALK	377-384	60.60%
		ETPGDWSHIIK	387-397	60.60%
		EYLPITGLADFNK	117-129	60.60%
		IRETPGDWSHIIK	385-397	60.60%
		ISMAGLSSR	429-437	60.60%
		LIFGADSPAIQENR	134-147	60.60%
		LVIRPMYSNPPLHGASIVATILK	331-353	60.60%
		MFVADGGELLMAQSYAK	284-300	60.60%
		NMGLYGER	301-308	60.60%
		QEYHIYMTSDGR	417-428	60.60%
		TEEGKPLVLNVVK	90-108	60.60%
		TIYIPQPTWGNHPK	175-188	60.60%
		TVPHLADAIHAAVTK	438-452	60.60%
		VATVQCLSGTGSLR	148-161	60.60%
		VFTLAGLTAR	189-198	60.60%
		VGALSIVCGSADIAVK	309-324	60.60%
		VGGEFLAR	162-169	60.60%
		VKEYLPITGLADFNK	115-129	60.60%
		VNLGVGAYR	81-89	60.60%
		YYDPATR	202-208	60.60%
TB3	Globulin-3A [Triticum aestivum]	AFVVPGLTDADGVGYVAQGEGVLTVIENGEK	182-212	42.50%
		ALRPFDEVSR	154-163	42.50%
		ASEEQLR	307-313	42.50%
		DTFNLLEQR	339-347	42.50%
		DTFNLLEQRPK	339-349	42.50%
		FQYFSAKPLLASLSK	257-271	42.50%
		GSAFVVPPGHPVVEIASSR	470-488	42.50%
		HEQEEQGR	74-81	42.50%
		ILHTISVPGK	247-256	42.50%
		LGSLLGSR	285-292	42.50%
		QASEGDQGHHWPLPPFR	318-334	42.50%
		QGDVIVAPAGSIMHLANTDGR	219-239	42.50%

		QGDVIVAPAGSIMHLANTDGRR	219-240	42.50%
		QGKEEEEK	293-300	42.50%
		REQEHGK	421-427	42.50%
		RPYVFGPR	132-139	42.50%
		RQASEGDQGHHWPLPPFR	317-334	42.50%
		RVLTAALK	272-279	42.50%
		SDHGFVK	147-153	42.50%
		SFHALAQHDVR	364-374	42.50%
		VAIMEVNPR	173-181	42.50%
		VAVANITPGSMTAPYLNTQSFK	375-396	42.50%
		WRSEEEEDDRR	430-440	42.50%
TB4	Globulin 1 [Triticum aestivum]	CAALRGMVR	90-98	80.00%
		CAALRGMVRDYEQSMPPLGEGR	90-111	80.00%
		CCQQLESVSR	77-86	80.00%
		CCQQLESVSRECR	77-89	80.00%
		DYEQSMPPLGEGR	99-111	80.00%
		EKPLHACR	39-46	80.00%
		EKPLHACRQILEQQLTGR	39-56	80.00%
		ERCCQQLESVSR	75-86	80.00%
		ERCCQQLESVSRECR	75-89	80.00%
		GCSGESTAEQR	123-133	80.00%
TB4	Globulin 1 [Triticum aestivum]	GCSGESTAEQRQEVQGGQYGSETGGSQQQ-GGGYHGVTVGR	123-162	80.00%
		GEVREKPLHACR	35-46	80.00%
		GGQRQGQVLCHK	163-174	80.00%
		GMVRDYEQSMPPLGEGR	95-111	80.00%
		HGSSGER	112-118	80.00%
		HGSSGERQPER	112-122	80.00%
		HGSSGERQPERGCSGESTAEQR	112-133	80.00%
		IEPQECSVFFADQYY	211-225	80.00%
		LADAQCR	28-34	80.00%
		LADAQCRGEVR	28-38	80.00%
		LADAQCRGEVREK	28-40	80.00%
		LPTACR	205-210	80.00%

		LPTACRIEPQECSVFFADQYY	205-225	80.00%
		LTKVRLPTACR	200-210	80.00%
		QEVQGGQYGSETGGSQQQGGGYHGVTVGR	134-162	80.00%
		QGQVLCHK	167-174	80.00%
		QGQVLCHKRPQR	167-178	80.00%
		QILEQQLTGR	47-56	80.00%
		QLESVSR	80-86	80.00%
		QLESVSRECR	80-89	80.00%
		QPERGCSGESTAEQR	119-133	80.00%
		QPERGCSGESTAEQRQEVQGGQYGSETGGSQQQ-GGGYHGVTVGR	119-162	80.00%
		QQGEGFSGEGAQQKPQAGR	179-197	80.00%
		QQGEGFSGEGAQQKPQAGRVR	179-199	80.00%
		VRLPTACR	203-210	80.00%
		VRLPTACRIEPQECSVFFADQYY	203-225	80.00%
		VRLTKVR	198-204	80.00%
TB4	Globulin-3A [Triticum aestivum]	AFVVPGLTDADGVGYVAQGEGVLTVIENGEK	182-212	65.50%
		AFVVPGLTDADGVGYVAQGEGVLTVIENGEKR	182-213	65.50%
		AKDQQDEGFVAGPEQQQEHER	542-562	65.50%
		AKDQQDEGFVAGPEQQQEHERGDR	542-565	65.50%
TB4	Globulin-3A [Triticum aestivum]	ALRPFDEVSR	154-163	65.50%
		ALRPFDEVSRLLR	154-166	65.50%
		ASEEQLR	307-313	65.50%
		ASEEQLRELR	307-316	65.50%
		ASEEQLRELRR	307-317	65.50%
		CQQDRPR	48-54	65.50%
		DSERREQEHGK	417-427	65.50%
		DTFNLLEQRPK	339-349	65.50%
		EVQEVFR	535-541	65.50%
		FQYFSAKPLLASLSK	257-271	65.50%
		GDSRDTFNLLEQRPK	335-349	65.50%
		GIRNYR	167-172	65.50%
		GSAFVVPPGHPVVEIASSR	470-488	65.50%
		GSGSESEEEQDQQRYETVR	446-464	65.50%

		GSSNLQVVCFEINAERNER	489-507	65.50%
		GSSNLQVVCFEINAERNERVWLAGR	489-513	65.50%
		HEQEEQGRGHGR	74-85	65.50%
		IANRHGR	350-356	65.50%
		IIRSDHGFVK	144-153	65.50%
		ILHTISVPGK	247-256	65.50%
		ILHTISVPGKFQYFSAKPLLASLSK	247-271	65.50%
		LDDPAQELAFGRPAR	520-534	65.50%
		LGSLLGSR	285-292	65.50%
		LLRGIR	164-169	65.50%
		LLRGIRNYR	164-172	65.50%
		LYEADAR	357-363	65.50%
		LYEADARSFHALAQHDVR	357-374	65.50%
		NNVIAKLDDPAQELAFGRPAR	514-534	65.50%
		NYRVAIMEVNPR	170-181	65.50%
		QGDVIVAPAGSIMHLANTDGR	219-239	65.50%
		QGDVIVAPAGSIMHLANTDGRR	219-240	65.50%
		QGKEEEEKSISIVR	293-306	65.50%
		REQEHGKGR	421-429	65.50%
TB4	Globulin-3A [Triticum aestivum]	RGQGEREEEQGR	102-113	65.50%
		RGSGSESEEEQDQQRYETVR	445-464	65.50%
		RKLVIAK	240-246	65.50%
		RPYVFGPR	132-139	65.50%
		RSYTVR	213-218	65.50%
		RVLTAALK	272-279	65.50%
		SDHGFVK	147-153	65.50%
		SFHALAQHDVR	364-374	65.50%
		TSDERLGSLLGSR	280-292	65.50%
		VAIMEVNPR	173-181	65.50%
		VLTAALK	273-279	65.50%
		VLTAALKTSDER	273-284	65.50%
		VLTAALKTSDERLGSLLGSR	273-292	65.50%
		VSRGSAFVVPPGHPVVEIASSR	467-488	65.50%
		VWLAGR	508-513	65.50%

		VWLAGRNNVIAK	508-519	65.50%
TB4	Alpha amylase inhibitor protein [Triticum aestivum]	CEALRYFIALPVPSQPVDPR	96-115	78.60%
		EMQWDFVR	133-140	78.60%
		EMQWDFVRLLVAPGQCNLATIHNVR	133-157	78.60%
		LLVAPGQCNLATIHNVR	141-157	78.60%
		LPEWMTSASIYSPGKPYLAK	61-80	78.60%
		LYCCQELAEISQQCR	81-95	78.60%
		LYCCQELAEISQQCRCEALR	81-100	78.60%
		LYCCQELAEISQQCRCEALRYFIALPVPSQPVDPR	81-115	78.60%
		SGNVGESGLIDLPGCPR	116-132	78.60%
		SGNVGESGLIDLPGCPREMQWDFVR	116-140	78.60%
		TNLLPHCR	37-44	78.60%
		TNLLPHCRDYVLQQTCGTFTPGSK	37-60	78.60%
		YCPAVEQPLWI	158-168	78.60%
		YFIALPVPSQPVDPR	101-115	78.60%

TB4	Endogenous alpha-amylase/subtilisin inhibitor	AHGGGLTMAPGHGR	27-40	89.40%
		AHGGGLTMAPGHGRR	27-41	89.40%
		AYTTCVQSTEWHIDSELVSGR	85-105	89.40%
		AYTTCVQSTEWHIDSELVSGRR	85-106	89.40%
		CPLFVSQEADGQR	42-54	89.40%
		CPLFVSQEADGQRDGLPVR	42-60	89.40%
		DLKGGAWFLGATEPYHVVVFK	155-175	89.40%
		DLKGGAWFLGATEPYHVVVFKK	155-176	89.40%
		DPPPVHDTDGNELR	1-14	89.40%
		DPPPVHDTDGNELRADANYYVLPANR	1-26	89.40%
		DPSPSGRENAFRIEK	115-129	89.40%
		ENAFRIEK	122-129	89.40%
		GGAWFLGATEPYHVVVFK	158-175	89.40%
		HVITGPVR	107-1114	89.40%
		HVITGPVRDPSPSGR	107-121	89.40%
		HVITGPVRDPSPSGRENAFR	107-126	89.40%

		IAPHGGAPSDK	61-71	89.40%
		IAPHGGAPSDKIIR	61-74	89.40%
		LSTDVRISFR	75-84	89.40%
		RCPLFVSQEADGQR	41-54	89.40%
		RCPLFVSQEADGQRDGLPVR	41-60	89.40%
		YSGAEVHEYK	130-139	89.40%
TB4	Class II chitinase [Triticum aestivum]	AAGRVPGFGVITNIINGGIECGHGQDSR	207-234	92.50%
		AAGRVPGFGVITNIINGGIECGHGQDSRVADR	207-238	92.50%
		AIGVDLLSNPDLVATDPTVSFK	154-175	92.50%
		AIGVDLLSNPDLVATDPTVSFKTAMWFWMTA-QAPKPSSHAVITGQWSPSGADR	154-206	92.50%
		AQFDRMLLHR	32-41	92.50%
		ARGSVSSVVSR	21-31	92.50%
		EVAAFLAQTSHETTGGWATAPDGAFAWGYCFKQER	79-113	92.50%
		GAASDYCTPSAQWPCAPGKR	114-133	92.50%
		GFYTYDAFVAAAAAFPGFGTTGSADAQKR	50-78	92.50%
		GPIQLSHNYNYGPAGR	138-153	92.50%
TB4	Class II chitinase [Triticum aestivum]	GSVSSVVSR	23-31	92.50%
		IGFYKR	239-244	92.50%
		MLLHRNDGACQAK	37-49	92.50%
		NDGACQAK	42-49	92.50%
		NDGACQAKGFYTYDAFVAAAAAFPGFGTTGSADAQKR	42-78	92.50%
		RYCDILGVGYGDNLDCYNQRPFA	244-266	92.50%
		RYYGRGPIQLSHNYNYGPAGR	133-153	92.50%
		TAMWFWMTAQAPKPSSHAVITGQWSPSGADR	176-206	92.50%
		VADRIGFYK	235-243	92.50%
		VADRIGFYKR	235-244	92.50%
		VPGFGVITNIINGGIECGHGQDSR	211-234	92.50%
		VPGFGVITNIINGGIECGHGQDSRVADR	211-238	92.50%
		YCDILGVGYGDNLDCYNQRPFA	245-266	92.50%
		YYGRGPIQLSHNYNYGPAGR	134-153	92.50%
TB5	Globulin-3A [Triticum aestivum]	AFVVPGLTDADGVGYVAQGEGVLTVIENGEK	182-212	30.30%
		AFVVPGLTDADGVGYVAQGEGVLTVIENGEKR	182-213	30.30%
		ALRPFDEVSR	154-163	30.30%

		ASEEQLR	307-313	30.30%
		EVQEVFR	535-541	30.30%
		FQYFSAKPLLASLSK	257-271	30.30%
		GSAFVVPPGHPVVEIASSR	470-488	30.30%
		ILHTISVPGK	247-256	30.30%
		LGSLLGSR	285-292	30.30%
		LVIAKILHTISVPGK	242-256	30.30%
		PLLASLSK	264-271	30.30%
		QGDVIVAPAGSIMHLANTDGR	219-239	30.30%
		QGDVIVAPAGSIMHLANTDGRR	219-240	30.30%
		RPYVFGPR	132-139	30.30%
		RVLTAALK	272-279	30.30%
		SDHGFVK	147-153	30.30%
		SDHGFVKALRPFDEVSR	147-163	30.30%
		SFHALAQHDVR	364-374	30.30%
		VAIMEVNPR	173-181	30.30%
TB5	Globulin-3A [Triticum aestivum]	VLTAALK	273-279	30.30%
