# Supplementary material for: A Mouse-Based Method to Monitor Wheat Allergens in Novel Wheat Lines and Varieties Created by Crossbreeding: Proof-of-Concept Using Durum and A. tauschii Wheats
Source: Int J Mol Sci. 2022 Jun 10;23(12):6505. doi: 10.3390/ijms23126505 (PMC9224339; doi:10.3390/ijms23126505)
Supplement: Supplementary file 1 [file ijms-23-06505-s001.zip › Supl_TableS2_TN1-TN7.pdf]

Table S2. Peptides sequences, positions, and coverage for wheat allergens identified in native tetraploid durum wheat.

IgE-binding band	Protein	Sequence	Position	Percent coverage
TN1	Globulin-3A [Triticum aestivum]	AFVVPGLTDADGVGYVAQGEGVLTVIENGEK	182-212	49.80%
		AFVVPGLTDADGVGYVAQGEGVLTVIENGEKR	182-213	49.80%
		AKDQQDEGFVAGPEQQQEHER	542-562	49.80%
		ALRPFDEVSR	154-163	49.80%
		ASEEQLR	307-313	49.80%
		DTFNLLEQR	339-347	49.80%
		DTFNLLEQRPK	339-349	49.80%
		EVQEVFR	535-541	49.80%
		FQYFSAKPLLASLSK	257-271	49.80%
		FQYFSAKPLLASLSKR	257-272	49.80%
		GDSRDTFNLLEQRPK	335-349	49.80%
		GSAFVVPPGHPVVEIASSR	470-488	49.80%
		GSGSESEEEQDQQRYETVR	446-464	49.80%
		ILHTISVPGK	247-256	49.80%
		ILHTISVPGKFQYFSAK	247-263	49.80%
		ILHTISVPGKFQYFSAKPLLASLSK	247-271	49.80%
		LAVVLEGEGEVEIVCPHLGR	397-416	49.80%
		LDDPAQELAFGRPAR	520-534	49.80%
		LVIAKILHTISVPGK	242-256	49.80%
		PFDEVSR	157-163	49.80%
		PLLASLSK	264-271	49.80%
		PLLASLSKR	264-272	49.80%
		QGDVIVAPAGSIMHLANTDGR	219-239	49.80%
		QGDVIVAPAGSIMHLANTDGRR	219-240	49.80%
		RPYVFGPR	132-139	49.80%
		RVLTAALK	272-279	49.80%
		SDHGFVK	147-153	49.80%
		SFHALAQHDVR	364-374	49.80%
		VAIMEVNPR	173-181	49.80%

		VAVANITPGSMTAPYLNTQSFK	375-396	49.80%
TN1	Globulin-3A [Triticum aestivum]	VLTAALK	273-279	49.80%
		WRSEEEEDDRR	430-440	49.80%
TN1	Serpin [Triticum aestivum]	AEAQSVDFQTK	127-137	51.80%
		AVLLSASPPSDMDFIADHPFLFLIR	354-378	51.80%
		DILPAGSISNTTR	159-171	51.80%
		DQLVATLGEGEAER	62-75	51.80%
		EDTSGVVLFIGHVVNPLR	379-396	51.80%
		HLGLQLPFSDEADLSEMVDSPMPQGLR	301-327	51.80%
		ISFGIEASDLLK	289-300	51.80%
		ISSVFHK	328-334	51.80%
		LHALAEQVVQFVLADASYADSPR	76-98	51.80%
		LSAEPELLER	261-270	51.80%
		LSIAHQTR	11-18	51.80%
		LVLGNALYFK	172-181	51.80%
		TFVEVNETGTEAAAATIAK	335-353	51.80%
		VTTGLIK	152-158	51.80%
		VTTGLIKDILPAGSISNTTR	152-171	51.80%
		YKAEAQSVDFQTK	125-137	51.80%
		AETQSVDFQTK	127-137	56.00%
		CLGLQLPFGDEADFSEMVDSLMPQGLR	301-327	56.00%
		DQLVATLGTGK	62-72	56.00%
		EDISGVVLFMGHVVNPLLSS	379-398	56.00%
		IKDILPPGSIDNTTK	157-171	56.00%
		ISFGIEASDLLK	289-300	56.00%
		LASTISSNPK	23-32	56.00%
		LSAEPDFLER	261-270	56.00%
		LSIAHQTR	11-18	56.00%
		LVLANALYFK	172-181	56.00%
		NDYFYLLDGSSVQTPFMSSMDDQYLLSSDGLK	195-226	56.00%
		SAASNAAFSPVSLYSALSLLAAGAGSATR	33-61	56.00%
		VSSVFHQAFVEVNEQGTEAAASTAIK	328-353	56.00%
		YKAETQSVDFQTK	125-137	56.00%

TN1	Fructose-1,6-bisphosphate aldolase 12 [Triticum aestivum]	AGNVLPGIKVDK	95-106	63.40%
		ALNDQHVLLEGTLLKPNMVTPGSDSK	211-236	63.40%
		ALNDQHVLLEGTLLKPNMVTPGSDSKK	211-237	63.40%
		ALQQSTLK	299-306	63.40%
		ANSEATLGTYK	328-338	63.40%
		FASINVENVEDNRR	40-53	63.40%
		GGKPFVDILK	85-94	63.40%
		GILAADESTGTIGK	25-38	63.40%
		GILAADESTGTIGKR	25-39	63.40%
		GTIELAGTNGETTTQGFDDLGK	107-128	63.40%
		GTIELAGTNGETTTQGFDDLGKR	107-129	63.40%
		IGATEPSQLSIDQNAQGLAR	149-168	63.40%
		KVAPEVIAEYTVR	237-249	63.40%
		NAAYIGTPGK	15-24	63.40%
		TVPAAVPAIVFLSGGQSEEEATLNLNAMNK	254-283	63.40%
		VAPEVIAEYTVR	238-249	63.40%
		VDKGTIELAGTNGETTTQGFDDLGK	104-128	63.40%
		VDKGTIELAGTNGETTTQGFDDLGKR	104-129	63.40%
		YAIICQENGLVPIVEPEILVDGPHDIDR	169-196	63.40%
		YKDELIK	8-14	63.40%
TN2	Tritin [Triticum aestivum]	ADNLYWEGFK	79-88	56.70%
		AQVNGWQDLSEALLK	202-216	56.70%
		DLLGDTDKLTNVALGR	116-131	56.70%
		EAVTTLLLMVHEATR	160-174	56.70%
		FQTVSGFVAGVLHPK	175-189	56.70%
		LRNPGHSSHNRPVLPPIEPNVPPSR	34-58	56.70%
		LTNVALGR	124-131	56.70%
		NPGHSSHNRPVLPPIEPNVPPSR	36-58	56.70%
		PVLPPIEPNVPPSR	45-58	56.70%
		QQMADAVTALYGR	132-144	56.70%
		SSDGTWWELTPGLIPGATHVGFGGTYR	89-115	56.70%
		TSPASTGLTLATR	66-78	56.70%
		WFHIVLK	59-65	56.70%
	Serpin	EDISGVVLFMGHVVNPLLSS	379-398	40.50%

TN2	[Triticum aestivum]	IKDILPSGSVDNTTK	157-171	40.50%
		ISFETEASDLLK	289-300	40.50%
		LSAEPDFLER	261-270	40.50%
		LSIAHQTR	11-18	40.50%
		LVLANALYFK	172-181	40.50%
		NDYFYLPDGSSVQTPFMSSMDDQYLSSSDGLK	195-226	40.50%
		QFSMYILLPEAPGGLSNLAEK	240-260	40.50%
		VLKLPYK	227-233	40.50%
		VSSVFHQAFVEVNEQGTEAAASTAIK	328-353	40.50%
		AAEVTTQVNSWVEK	138-151	54.50%
		AETQSVDFQTK	127-137	54.50%
		EDISGVVLFMGHVVNPLLSS	379-398	54.50%
		IKDILPPGSIDNTTK	157-171	54.50%
		ISFGIEASDLLK	289-300	54.50%
		LSAEPDFLER	261-270	54.50%
		LSIAHQTR	11-18	54.50%
		LVLANALYFK	172-181	54.50%
		NDYFYLLDGSSVQTPFMSSMDDQYLLSSDGLK	195-226	54.50%
		QFFMYILLPEAPGGLSSLAEK	240-260	54.50%
		SAASNAAFSPVSLYSALSLLAAGAGSATR	33-61	54.50%
		VLKLPYK	227-233	54.50%
		VSSVFHQAFVEVNEQGTEAAASTAIK	328-353	54.50%
		YKAETQSVDFQTK	125-137	54.50%
TN2	Peroxidase 1 [Triticum aestivum]	AVNDIRDRLER	113-123-	50.80%
		DFFEQFGVSMGK	298309	50.80%
		DIGLAAGLLR	62-71	50.80%
		DSVVVSGGPDYR	142-153	50.80%
		DSVVVSGGPDYRVPLGR	142-158	50.80%
		EGLFVSDQDLFTNDITR	270-286	50.80%
		EGLFVSDQDLFTNDITRPIVER	270-291	50.80%
		GFVQDAVR	53-60	50.80%
		GFVQDAVRK	53-61	50.80%
TN2	Peroxidase 1 [Triticum aestivum]	GLSFDFYR	34-41	50.80%
		GLSFDFYRR	34-42	50.80%

		KDIGLAAGLLR	61-71	50.80%
		LFPRPDPTINPPFLAR	219-234	50.80%
		PDPTINPPFLAR	223-234	50.80%
		PLGLDATDLVTISGGHTIGQAHCSSFEDR	190-218	50.80%
		QDVLSDLPAPSSNVPSLLALLR	168-189	50.80%
		TPNVFDNQYYVDLVNR	254-269	50.80%
		TSDLGEVR	317-234	50.80%
		TSDLGEVRR	317-235	50.80%
TN3	Class II chitinase [Triticum aestivum]	AIGVDLLSNPDLVATDPTVSFK	154-175	99.70%
		GAASDYCTPSAQWPCAPGK	114-132	99.70%
		GFYTYDAFVAAAAAFPGFGTTGSADAQK	50-77	99.70%
		GFYTYDAFVAAAAAFPGFGTTGSADAQKR	50-78	99.70%
		GPIQLSHNYNYGPAGR	138-153	99.70%
		GSVSSVVSR	23-31	99.70%
		PSSHAVITGQWSPSGADR	189-206	99.70%
		RYCDILGVGYGDNLDCYNQRPFA	244-266	99.70%
		VPGFGVITNIINGGIECGHGQDSR	211-234	99.70%
		YCDILGVGYGDNLDCYNQR	245-263	99.70%
		YCDILGVGYGDNLDCYNQRPFA	245-266	99.70%
TN4	Serpin [Triticum aestivum]	EDISGVVLFMGHVVNPLLSS	379-398	38.70%
		IKDILPSGSVDNTTK	157-171	38.70%
		ISFETEASDLLK	289-300	38.70%
		LSAEPDFLER	261-270	38.70%
		LSIAHQTR	11-18	38.70%
		LVLANALYFK	172-181	38.70%
		NDYFYLPDGSSVQTPFMSSMDDQYLSSSDGLK	195-226	38.70%
		QFSMYILLPEAPGGLSNLAEK	240-260	38.70%
		VSSVFHQAFVEVNEQGTEAAASTAIK	328-353	38.70%
		DQLVATLGTGK	62-72	52.00%
		EDISGVVLFMGHVVNPLLSS	379-398	52.00%
		IKDILPPGSIDNTTK	157-171	52.00%
TN4	Serpin [Triticum aestivum]	ISFGIEASDLLK	289-300	52.00%
		LSAEPDFLER	261-270	52.00%
		LSIAHQTR	11-18	52.00%

		LVLANALYFK	172-181	52.00%
		NDYFYLLDGSSVQTPFMSSMDDQYLLSSDGLK	195-226	52.00%
		QFFMYILLPEAPGGLSSLAEK	240-260	52.00%
		SAASNAAFSPVSLYSALSLLAAGAGSATR	33-61	52.00%
		VSSVFHQAFVEVNEQGTEAAASTAIK	328-353	52.00%
		YKAETQSVDFQTK	125-137	52.00%
TN4	Dehydroascorbate reductase [Triticum aestivum]	AAVGHPDTLGDCPFSQR	8-24	86.30%
		AHGPYINGANISAVDLSLAPK	136-156	86.30%
		ALVDELQALEEHLK	122-135	86.30%
		ENLIAGWAPK	200-209	86.30%
		IFSTFVTFLK	102-111	86.30%
		KVPYQMK	33-39	86.30%
		LIDVSNKPDWFLK	40-52	86.30%
		LYHLQVALEHFK	157-168	86.30%
		SKDASDGSEK	112-121	86.30%
		VLLTLEEK	25-32	86.30%
		VPETLTSVHAYTEALFSR	172-189	86.30%
		VPVYNGGDGK	59-68	86.30%
		WIADSDVITQVIEEK	69-83	86.30%
		YPTPSLVTPAEYASVGSK	84-101	86.30%
TN4	Endogenous alpha-amylase/subtilisin inhibitor	ADANYYVLPANR	15-26	80.60%
		AHGGGLTMAPGHGR	27-40	80.60%
		AHGGGLTMAPGHGRR	27-41	80.60%
		AYTTCVQSTEWHIDSELVSGR	85-105	80.60%
		CPLFVSQEADGQR	42-54	80.60%
		DPPPVHDTDGNELR	1-14	80.60%
		DPPPVHDTDGNELRADANYYVLPANR	1-26	80.60%
		GGAWFLGATEPYHVVVFK	158-175	80.60%
		HVITGPVR	107-114	80.60%
		HVITGPVRDPSPSGR	107-121	80.60%
TN4	Endogenous alpha-amylase/subtilisin inhibitor	IAPHGGAPSDK	61-71	80.60%
		LMACGDSCQDLGVFR	140-154	80.60%
		RCPLFVSQEADGQR	41-54	80.60%
		RHVITGPVRDPSPSGR	106-121	80.60%

		YSGAEVHEYK	130-139	80.60%
TN4	Glyceraldehyde-3-phosphate dehydrogenase [Triticum aestivum]	AAIKEESEGNLK	262-273	49.20%
		AASFNIIPSSTGAAK	203-217	49.20%
		DAPMFVCGVNEK	129-140	49.20%
		DAPMFVCGVNEKEYK	129-143	49.20%
		FGIVEGLMTTVHAMTATQK	170-188	49.20%
		GILGYVDEDLVSTDFQGDNR	274-293	49.20%
		IGINGFGR	6-13	49.20%
		KVIISAPSK	120-128	49.20%
		SDIDIVSNASCTTNCLAPLAK	144-164	49.20%
		SSIFDAK	294-300	49.20%
		VALQSPDVELVAVNDPFITTDYMTYMFK	21-48	49.20%
		VLPELNGK	222-229	49.20%
		VPTVDVSVVDLTVR	237-250	49.20%
		AASFNIIPSSTGAAK	202-216	47.80%
		AASYDDIKK	253-261	47.80%
		AGIALNDHFVK	300-310	47.80%
		DAPMFVVGVNEDK	128-140	47.80%
		GIMGYVEEDLVSTDFVGDSR	273-292	47.80%
		IGINGFGR	5-12	47.80%
		IINDNFGIIEGLMTTVHAITATQK	164-187	47.80%
		KVVISAPSK	119-127	47.80%
		LTGMSFR	229-235	47.80%
		SSIFDAK	293-299	47.80%
		TLLFGEKPVTVFGVR	67-81	47.80%
		VLPELNGK	221-228	47.80%
		VPTVDVSVVDLTVR	236-249	47.80%

TN5	Endogenous alpha-amylase/subtilisin inhibitor	AHGGGLTMAPGHGR	27-40	69.40%
		AHGGGLTMAPGHGRR	27-41	69.40%
		CPLFVSQEADGQR	42-54	69.40%
		DPPPVHDTDGNELR	1-14	69.40%

		DPPPVHDTDGNELRADANYYVLPANR	1-26	69.40%
		GGAWFLGATEPYHVVVFK	158-175	69.40%
		GGAWFLGATEPYHVVVFKK	158-176	69.40%
		HVITGPVR	107-114	69.40%
		HVITGPVRDPSPSGR	107-121	69.40%
		IAPHGGAPSDK	61-71	69.40%
		LMACGDSCQDLGVFR	140-154	69.40%
		RCPLFVSQEADGQR	41-54	69.40%
		RHVITGPVRDPSPSGR	106-121	69.40%
		YSGAEVHEYK	130-139	69.40%
TN5	Globulin-3A [Triticum aestivum]	AFVVPGLTDADGVGYVAQGEGVLTVIENGEK	182-212	41.30%
		AKDQQDEGFVAGPEQQQEHER	542-562	41.30%
		AKDQQDEGFVAGPEQQQEHERGDR	542-565	41.30%
		ALRPFDEVSR	154-163	41.30%
		ASEEQLR	307-313	41.30%
		DQQDEGFVAGPEQQQEHER	544-562	41.30%
		DTFNLLEQRPK	339-349	41.30%
		EVQEVFR	535-541	41.30%
		FQYFSAK	257-263	41.30%
		FQYFSAKPLLASLSK	257-271	41.30%
		GSAFVVPPGHPVVEIASSR	470-488	41.30%
		GSGSESEEEQDQQR	446-459	41.30%
		GSGSESEEEQDQQRYETVR	446-464	41.30%
		GSSNLQVVCFEINAER	489-504	41.30%
		ILHTISVPGK	247-256	41.30%
		LDDPAQELAFGR	520-531	41.30%
		LDDPAQELAFGRPAR	520-534	41.30%
		PLLASLSK	264-271	41.30%
TN5	Globulin-3A [Triticum aestivum]	QGDVIVAPAGSIMHLANTDGR	219-239	41.30%
		QGDVIVAPAGSIMHLANTDGRR	219-240	41.30%
		RGSGSESEEEQDQQRYETVR	445-464	41.30%
		RPYVFGPR	132-139	41.30%
		RVLTAALK	272-279	41.30%
		SFHALAQHDVR	364-374	41.30%

		VAIMEVNPR	173-181	41.30%
		VLTAALK	273-279	41.30%
TN6	Globulin-3A [Triticum aestivum]	AFVVPGLTDADGVGYVAQGEGVLTVIENGEK	182-212	40.80%
		AKDQQDEGFVAGPEQQQEHER	542-562	40.80%
		AKDQQDEGFVAGPEQQQEHERGDR	542-565	40.80%
		ALRPFDEVSR	154-163	40.80%
		ASEEQLR	307-313	40.80%
		DQQDEGFVAGPEQQQEHER	544-562	40.80%
		DQQDEGFVAGPEQQQEHERGDR	544-565	40.80%
		DTFNLLEQR	339-347	40.80%
		DTFNLLEQRPK	339-349	40.80%
		EVQEVFR	535-541	40.80%
		GRGDEAVEAFLR	571-582	40.80%
		GSAFVVPPGHPVVEIASSR	470-488	40.80%
		GSGSESEEEQDQQR	446-459	40.80%
		GSGSESEEEQDQQRYETVR	446-464	40.80%
		GSSNLQVVCFEINAER	489-504	40.80%
		GSSNLQVVCFEINAERNER	489-507	40.80%
		ILHTISVPGK	247-256	40.80%
		LDDPAQELAFGR	520-531	40.80%
		LDDPAQELAFGRPAR	520-534	40.80%
		LGSLLGSR	285-292	40.80%
		LYEADAR	357-363	40.80%
		QGDVIVAPAGSIMHLANTDGR	219-239	40.80%
		RGSGSESEEEQDQQR	445-459	40.80%
		RGSGSESEEEQDQQRYETVR	445-464	40.80%
		RPYVFGPR	132-139	40.80%
TN6	Globulin-3A [Triticum aestivum]	SFHALAQHDVR	364-374	40.80%
TN6	Heat shock protein 17.3 [Triticum aestivum]	AMAATPADVK	48-57	47.80%
		DGVLTVTVDK	134-143	47.80%
		DGVLTVTVDKLPPPEPK	134-150	47.80%
		ELPGAYAFVVDMPGLGSGDIK	58-78	47.80%
		FVLPENADMEK	117-127	47.80%

		KFVLPENADMEK	116-127	47.80%
		LPPPEPK	144-150	47.80%
		VLVISGER	86-93	47.80%
		VQVEDER	79-85	47.80%
TN6	Alpha amylase inhibitor protein [Triticum aestivum]	DYVLQQTCGTFTPGSK	45-60	69.00%
		EMQWDFVR	133-140	69.00%
		LLVAPGQCNLATIHNVR	141-157	69.00%
		LPEWMTSASIYSPGKPYLAK	61-80	69.00%
		LYCCQELAEISQQCR	81-95	69.00%
		SGNVGESGLIDLPGCPR	116-132	69.00%
		TNLLPHCR	37-44	69.00%
		YFIALPVPSQPVDPR	101-115	69.00%
TN7	Histone H4 [Triticum aestivum]	DAVTYTEHAR	69-78	59.20%
		DNIQGITK	25-32	59.20%
		DNIQGITKPAIR	25-36	59.20%
		ISGLIYEETR	47-56	59.20%
		ISGLIYEETRGVLK	47-60	59.20%
		KTVTAMDVVYALK	80-92	59.20%
		KTVTAMDVVYALKR	80-93	59.20%
		RISGLIYEETR	46-56	59.20%
		TLYGFGG	97-103	59.20%
		TVTAMDVVYALK	81-92	59.20%
		TVTAMDVVYALKR	81-93	59.20%
		VLRDNIQGITKPAIR	22-36	59.20%
TN7	Glyceraldehyde-3-phosphate dehydrogenase [Triticum aestivum]	AASYDDIK	253-260	55.50%
		AASYDDIKK	253-261	55.50%
		AGIALNDHFVK	300-310	55.50%
		GIMGYVEEDLVSTDFVGDSR	273-292	55.50%
		IINDNFGIIEGLMTTVHAITATQK	164-187	55.50%
		KVVISAPSK	119-127	55.50%
		LTGMSFR	229-235	55.50%

		NPEEIPWGEAGADYVVESTGVFTDKDK	82-108	55.50%
		SSIFDAK	293-299	55.50%
		TLLFGEK	67-73	55.50%
		TLLFGEKPVTVFGVR	67-81	55.50%
		VALQSDDVELVAVNDPFITTEYMTYMFK	20-47	55.50%
		VPTVDVSVVDLTVR	236-249	55.50%
		AAIKEESEGNLK	262-27	57.80%
		AASFNIIPSSTGAAK	203-217	57.80%
		AGIALNDNFVK	301-311	57.80%
		DAPMFVCGVNEK	129-140	57.80%
		EVAVFGCR	75-82	57.80%
		FGIVEGLMTTVHAMTATQK	170-188	57.80%
		GILGYVDEDLVSTDFQGDNR	274-293	57.80%
		KVIISAPSK	120-128	57.80%
		LAKPATYDQIK	251-261	57.80%
		LTGMAFR	230-236	57.80%
		NPEEIPWAAAGAEYVVESTGVFTDKDK	83-109	57.80%
		PATYDQIK	254-261	57.80%
		SSIFDAK	294-300	57.80%
		TLLFGEK	68-74	57.80%
		VALQSPDVELVAVNDPFITTDYMTYMFK	21-48	57.80%
		VIISAPSK	121-128	57.80%
		VPTVDVSVVDLTVR	237-250	57.80%
