# Supplementary material for: A Mouse-Based Method to Monitor Wheat Allergens in Novel Wheat Lines and Varieties Created by Crossbreeding: Proof-of-Concept Using Durum and A. tauschii Wheats
Source: Int J Mol Sci. 2022 Jun 10;23(12):6505. doi: 10.3390/ijms23126505 (PMC9224339; doi:10.3390/ijms23126505)
Supplement: Supplementary file 1 [file ijms-23-06505-s001.zip › Supl_TableS3_DB1-DB2.pdf]

Table S3. Peptides sequences, positions, and coverage for wheat allergens identified in boiled/reduced tauschii wheat.

IgE-binding band	Protein	Sequence	Position	Percent coverage
DB1	Serpin 3 [Triticum aestivum]	AAEVTTQVNSWVEK	138-151	91.70%
		AAFSPVSLHSALSLLAAGAGSATR	38-61	91.70%
		AETQSVDFQTK	127-137	91.70%
		AFSPVSLHSALSLLAAGAGSATR	39-61	91.70%
		AFVEVNEQGTEAAASTAIK	336-354	91.70%
		ASNAAFSPVSLHSALSLLAAGAGSATR	35-61	91.70%
		ATTLATDVR	2-10	91.70%
		AWTDQFDSYGTK	183-194	91.70%
		CLGLQLPFSDEADFSEMVDSPMPQGLR	302-328	91.70%
		DDQYISSSDGLK	216-227	91.70%
		DQLVATLGTGEVEGLHALAEQVVQF	62-86	91.70%
		DQLVATLGTGEVEGLHALAEQVVQFVLADASSAGGPR	62-98	91.70%
		EDISGVVLF	380-388	91.70%
		EDISGVVLFMGHVVN	380-394	91.70%
		EDISGVVLFMGHVVNPL	380-396	91.70%
		EPDFLER	265-271	91.70%
		EVTTQVNSWVEK	140-151	91.70%
		FGIEASDLLK	292-301	91.70%
		FSPVSLHSALSLLAAGAGSATR	40-61	91.70%
		FVLADASSAGGPR	86-98	91.70%
		GAWTDQFD	182-189	91.70%
		GAWTDQFDSYGTK	182-194	91.70%
		HSALSLLAAGAGSATR	46-61	91.70%
		IKNILPSGSVDN	157-168	91.70%
		IKNILPSGSVDNTTK	157-171	91.70%
		IKNILPSGSVDNTTKLVLANALYFK	157-181	91.70%
		ILLPEAPGGLSSLAEK	246-261	91.70%
		ILPSGSVDNTTK	160-171	91.70%
		ISFGIEASDLLK	290-301	91.70%
		KAETQSVDFQTK	126-137	91.70%

		LANALYFK	174-181	91.70%
		LASTISSNPK	23-32	91.70%
DB1	Serpin 3 [Triticum aestivum]	LKPSFQELAVCK	113-124	91.70%
		LPEAPGGLSSLAEK	248-261	91.70%
		LSAEPDFLER	262-271	91.70%
		LSAEPDFLERHIPR	262-275	91.70%
		LSIAHQTR	11-18	91.70%
		LSLLAAGAGSATR	49-61	91.70%
		LVLANAL	172-178	91.70%
		LVLANALYFK	172-181	91.70%
		MSSMDDDQYISSSDGLK	211-227	91.70%
		MVPQQARPPSVMDFIADHPFLFLLR	355-379	91.70%
		NAAFSPVSLHSALSLLAAGAGSATR	37-61	91.70%
		NDYFYLLDGSSVQTPF	195-178	91.70%
		NDYFYLLDGSSVQTPFMSSMDD	195-181	91.70%
		NDYFYLLDGSSVQTPFMSSMDDDQYISSSDGLK	195-227	91.70%
		NILPSGSVDNTTK	159-171	91.70%
		PEAPGGLSSLAEK	249-261	91.70%
		PGGLSSLAEK	252-261	91.70%
		PPSVMDFIADHPFLFLLR	362-379	91.70%
		PQQARPPSVMDFIADHPFLFLLR	357-379	91.70%
		PSFQELAVCK	115-124	91.70%
		PSGSVDNTTK	162-171	91.70%
		QARPPSVMDFIADHPFLFLLR	359-379	91.70%
		QFSMYILLPEAPGGLSSLA	241-259	91.70%
		QFSMYILLPEAPGGLSSLAEK	241-261	91.70%
		QSVDFQTK	130-137	91.70%
		QYISSSDGLK	218-227	91.70%
		SAASNAAFSPVSLH	33-46	91.70%
		SAASNAAFSPVSLHS	33-47	91.70%
		SAASNAAFSPVSLHSA	33-48	91.70%
		SAASNAAFSPVSLHSAL	33-49	91.70%
		SAASNAAFSPVSLHSALSLLAAGAGSA	33-59	91.70%
		SAASNAAFSPVSLHSALSLLAAGAGSATR	33-61	91.70%

		SAEPDFLERHIPR	263-275	91.70%
DB1	Serpin 3 [Triticum aestivum]	SALSLLAAGAGSATR	47-61	91.70%
		SIAHQTR	12-18	91.70%
		SLLAAGAGSATR	50-61	91.70%
		SPVSLHSALSLLAAGAGSATR	41-61	91.70%
		TTLATDVR	3-10	91.70%
		VAFANGVFVDASLLLK	99-114	91.70%
		VAFANGVFVDASLLLKPSFQELAVCK	99-124	91.70%
		VLADASSAGGPR	87-98	91.70%
		VLANALYFK	173-181	91.70%
		VLKLPYK	228-234	91.70%
		VSSVFHQAFVEVN	329-341	91.70%
		VSSVFHQAFVEVNEQ	329-343	91.70%
		VSSVFHQAFVEVNEQGTEAAA	329-349	91.70%
		VSSVFHQAFVEVNEQGTEAAAST	329-351	91.70%
		VSSVFHQAFVEVNEQGTEAAASTAIK	329-354	91.70%
		YILLPEAPGGLSSLAEK	245-261	91.70%
		YKAETQSVDFQTK	125-137	91.70%
		YKAETQSVDFQTKAAEVTTQVNSWVEK	125-151	91.70%
DB1	Serpin-N3.2 [Triticum aestivum]	AAEVTAQVNSWVEK	138-151	93.00%
		AAEVTAQVNSWVEKVTTGLIK	138-158	93.00%
		AEAQSVDFQTK	127-137	93.00%
		AEAQSVDFQTKAAEVTAQVNSWVEK	127-151	93.00%
		AEPEFLEQHIPR	263-274	93.00%
		AFVEVNETGTEAAATTIAK	335-353	93.00%
		ALAEQVVQFVLADASNIGGPR	78-98	93.00%
		ATQSDDFYLLD	192-202	93.00%
		ATQSDDFYLLDGSSIQTPF	192-210	93.00%
		ATQSDDFYLLDGSSIQTPFMY	192-212	93.00%
		ATQSDDFYLLDGSSIQTPFMYSSEEQYISSSDGLK	192-226	93.00%
		ATTLATDVR	2-10	93.00%
		DGSSIQTPFMYSSEEQYISSSDGLK	202-226	93.00%
		DILPAGSIDNTTR	159-171	93.00%
		EAQSVDFQTK	128-137	93.00%

DB1	Serpin-N3.2 [Triticum aestivum]	EDTSGVVLFIGH	379-390	93.00%
		EDTSGVVLFIGHVVN	379-393	93.00%
		EDTSGVVLFIGHVVNPL	379-395	93.00%
		EDTSGVVLFIGHVVNPLLS	379-397	93.00%
		EDTSGVVLFIGHVVNPLLSS	379-398	93.00%
		EFLEQHIPR	266-274	93.00%
		EPEFLEQHIPR	264-274	93.00%
		EVTAQVNSWVEK	140-151	93.00%
		GAWTDQFD	182-189	93.00%
		GAWTDQFDPR	182-191	93.00%
		GHVVNPLLSS	389-398	93.00%
		GLGLQLPF	301-308	93.00%
		GLGLQLPFGAEADLSEMVDSPMAQNLYISSIFHK	301-334	93.00%
		HVALSLITAGAGGATR	46-61	93.00%
		IGHVVNPLLSS	388-398	93.00%
		IKDILPAGSIDNTTR	157-171	93.00%
		ILLPEALSGLWSLAEK	245-260	93.00%
		ILPAGSIDNTTR	160-171	93.00%
		ISLGIEASDLLK	289-300	93.00%
		KAEAQSVDFQTK	126-137	93.00%
		LASAISSNPESTVN	23-36	93.00%
		LASAISSNPESTVNNAAFSPVSL	23-45	93.00%
		LASAISSNPESTVNNAAFSPVSLH	23-46	93.00%
		LASAISSNPESTVNNAAFSPVSLHVAL	23-49	93.00%
		LASAISSNPESTVNNAAFSPVSLHVALSLITAGAGGA	23-59	93.00%
		LASAISSNPESTVNNAAFSPVSLHVALSLITAGAGGATR	23-61	93.00%
		LGNALYFK	174-181	93.00%
		LKPSFQELAVCK	113-124	93.00%
		LPAGSIDNTTR	161-171	93.00%
		LPEALSGLWSLAEK	247-260	93.00%
		LSAEPEFLEQHIPR	261-274	93.00%
		LSIAHQTR	11-18	93.00%
		LSLITAGAGGATR	49-61	93.00%
	Serpin-N3.2	LVLGNALYFK	172-181	93.00%

DB1	[Triticum aestivum]	LYISSIFHK	326-334	93.00%
		MYSSEEQYISSSDGLK	211-226	93.00%
		NAAFSPVSLHVALSLITAGAGGATR	37-61	93.00%
		NLYISSIFHK	325-334	93.00%
		NQLAATLGEGEVEGLHALAEQVVQF	62-86	93.00%
		NQLAATLGEGEVEGLHALAEQVVQFVLADASN	62-92	93.00%
		NQLAATLGEGEVEGLHALAEQVVQFVLADASNIGGPR	62-98	93.00%
		PAGSIDNTTR	162-171	93.00%
		PEALSGLWSLAEK	248-260	93.00%
		PEFLEQHIPR	265-274	93.00%
		PESTVNNAAFSPVSLHVALSLITAGAGGATR	31-61	93.00%
		PPPSVLDFIVDHPFLFLIR	360-378	93.00%
		PSFQELAVCK	115-124	93.00%
		PSVLDFIVDHPFLFLIR	362-378	93.00%
		QAPPPSVLDFIVDHPF	358-373	93.00%
		QAPPPSVLDFIVDHPFLF	358-375	93.00%
		QAPPPSVLDFIVDHPFLFLIR	358-378	93.00%
		QFSMYILLPEALSGLWSLAEK	240-260	93.00%
		QLAATLGEGEVEGLHALAEQVVQFVLADASNIGGPR	63-98	93.00%
		QLKPSFQELAVCK	112-124	93.00%
		QSVDFQTK	130-137	93.00%
		QYISSSDGLK	217-226	93.00%
		RQFSMYILLPEALSGLWSLAEK	239-260	93.00%
		SAEPEFLEQHIPR	262-274	93.00%
		SIAHQTR	12-18	93.00%
		SPMAQNLYISSIFHK	320-334	93.00%
		SSEEQYISSSDGLK	213-226	93.00%
		TLGEGEVEGLHALAEQVVQFVLADASNIGGPR	67-98	93.00%
		TTLATDVR	3-10	93.00%
		VAFANGVFVDASLQLKPSFQELAVCK	99-124	93.00%
		VALSLITAGAGGATR	47-61	93.00%
		VLADASNIGGPR	87-98	93.00%
DB1	Serpin-N3.2 [Triticum aestivum]	VLKLPYK	227-233	93.00%
		VTTGLIK	152-158	93.00%

		VTTGLIKDILPAGSIDNTTR	152-171	93.00%
		VVNPLLSS	391-398	93.00%
		YILLPEALSGLWSLAEK	244-260	93.00%
		YKAEAQSVDFQTK	125-137	93.00%
		YSSEEQYISSSDGLK	212-226	93.00%
DB1	Globulin 3 [Triticum aestivum]	AFVVPGLTDADGVGYVAQGEGVLTVIENGEK	182-212	33.80%
		AFVVPGLTDADGVGYVAQGEGVLTVIENGEKR	182-213	33.80%
		ALRPFDEVSR	154-163	33.80%
		DTFNLLEQRPK	339-349	33.80%
		EIASSRGSSNLQVVCFEINAER	483-504	33.80%
		EVQEVFR	535-541	33.80%
		FHALAQHDVR	365-374	33.80%
		FNLLEQRPK	341-349	33.80%
		FQYFSAK	257-263	33.80%
		FQYFSAKPLLASLSK	257-271	33.80%
		GVGYVAQGEGVLTVIENGEK	193-212	33.80%
		GVGYVAQGEGVLTVIENGEKR	193-213	33.80%
		HALAQHDVR	366-374	33.80%
		HGQGEREEEQGR	102-113	33.80%
		LAVVLEGEGEVEIVCPHLGR	397-416	33.80%
		LYEADAR	357-363	33.80%
		NLLEQRPK	342-349	33.80%
		PLLASLSK	264-271	33.80%
		PLPPFR	329-334	33.80%
		QGDVIVAPAGSIMHLANTDGR	219-239	33.80%
		QGDVIVAPAGSIMHLANTDGRR	219-240	33.80%
		RPYVFGPR	132-139	33.80%
		SAKPLLASLSK	261-271	33.80%
		SDHGFVK	147-153	33.80%
		SFHALAQHDVR	364-374	33.80%
		TFNLLEQRPK	340-349	33.80%
DB1	Globulin 3 [Triticum aestivum]	VAIMEVNPR	173-181	33.80%
	Serpin 2	APEIASQVNSWVENVTTGLIR	141-161	85.60%

DB1	[Triticum aestivum]	ATDVCLSVAHQTR	6-18	85.60%
		ATTLATDVCLSVAHQTR	2-18	85.60%
		DDLWTLAK	255-262	85.60%
		DQLVAILGNENAGGPEGLHSLAEQVVQLVLADASITGDPR	62-101	85.60%
		EANLSEMVNSQVDLFLSSVFHK	314-335	85.60%
		EIASQVNSWVENVTTGLIR	143-161	85.60%
		EILPEGSIDYTTR	162-174	85.60%
		FHLLNGNTVQTPF	201-213	85.60%
		FHLLNGNTVQTPFMSSTNK	201-219	85.60%
		GLWTEKFDESK	185-195	85.60%
		ISFGFEATNLLK	292-303	85.60%
		LASAISSDPESATGNVAFSPVSLHVALSLITAGAGGTTR	23-61	85.60%
		LGNALYFK	177-184	85.60%
		LLPERR	249-254	85.60%
		LSTESEFIEK	264-273	85.60%
		LVLGNALYFK	175-184	85.60%
		QFSMYILLPER	243-253	85.60%
		QFSMYILLPERR	243-254	85.60%
		QYISSSDGLK	220-229	85.60%
		RDDLWTLAK	254-262	85.60%
		RLSTESEFIEK	263-273	85.60%
		SFVEVNEQGTEAAAATSVAIEQQQMPIVMDFVADHPF LFLIR	336-377	85.60%
		SLGLQLPFSR	304-313	85.60%
		TKYDKFHLLNGNTVQTPFMSSTNK	196-219	85.60%
		VAFANGVFVDASLSLKPSFQELAVCNYK	102-129	85.60%
		VVVDQFMLPK	280-289	85.60%
		YDKFHLLNGNTVQTPFMSSTNK	198-219	85.60%
DB2	Peroxidase 1 [Triticum aestivum]	AAEPPVAR	26-33	72.60%
DB2	Peroxidase 1 [Triticum aestivum]	ADILALAAR	133-141	72.60%
		ALLRPLGLDATDLVTISGGHTIGQAHCSSFEDR	186-218	72.60%
		APPNLTLRPSAFK	100-112	72.60%
		ASVLLDGSATGPGEQQAPPNLTLRPSAFK	84-112	72.60%

		ATGPGEQQAPPNLTLRPSAFK	92-112	72.60%
		AVNDIRDR	113-120	72.60%
		AVNDIRDRLER	113-123	72.60%
		AVVSCADILALAAR	128-141	72.60%
		CADILALAAR	132-141	72.60%
		DASVLLDGSATGPGEQQAPPNLTLRPSAFK	83-112	72.60%
		DFFEQFGVSMGK	298-309	72.60%
		DFFEQFGVSMGKMGQMR	298-314	72.60%
		DQDLFTNDITRPIVER	276-291	72.60%
		DSVVVSGGPDYR	142-153	72.60%
		DSVVVSGGPDYRVPLGR	142-158	72.60%
		EGLFVSDQDLFTNDITR	270-286	72.60%
		EGLFVSDQDLFTNDITRPIVER	270-291	72.60%
		EQQAPPNLTLRPSAFK	97-112	72.60%
		FEQFGVSMGK	300-309	72.60%
		FPRPDPTINPPFLAR	220-234	72.60%
		FVSDQDLFTNDITRPIVER	273-291	72.60%
		GAVVSCADILALAAR	127-141	72.60%
		GFVQDAVR	53-60	72.60%
		GFVQDAVRK	53-61	72.60%
		GLDATDLVTISGGHTIGQAHCSSFEDR	192-218	72.60%
		GLFVSDQDLFTNDITRPIVER	271-291	72.60%
		GSATGPGEQQAPPNLTLRPSAFK	90-112	72.60%
		KDIGLAAGLLR	61-71	72.60%
		LFPRPDPTIN	219-228	72.60%
		LFPRPDPTINPPFL	219-232	72.60%
		LFPRPDPTINPPFLAR	219-234	72.60%
		LHFHDCFVQG	72-81	72.60%
		LHFHDCFVQGCDASVLLDGSATGPGEQQAPPNLTLRPSAFK	72-112	72.60%
DB2	Peroxidase 1 [Triticum aestivum]	LRPLGLDATDLVTISGGHTIGQAHCSSFEDR	188-218	72.60%
		LSFDFYR	35-41	72.60%
		LTLRPSAFK	104-112	72.60%
		NVFDNQYYVDLVNR	256-269	72.60%
		PAPSSNVPSLLALLR	175-189	72.60%

		PDPTINPPFLAR	223-234	72.60%
		PGEQQAPPNLTLRPSAFK	95-112	72.60%
		PLGLDATDLVTISGGH	190-205	72.60%
		PLGLDATDLVTISGGHTIGQAH	190-211	72.60%
		PLGLDATDLVTISGGHTIGQAHCSSFEDR	190-218	72.60%
		PPNLTLRPSAFK	101-112	72.60%
		PRPDPTINPPFLAR	221-234	72.60%
		PSSNVPSLLALLR	177-189	72.60%
		QAPPNLTLRPSAFK	99-112	72.60%
		QDVLSDLPAPSSN	168-180	72.60%
		QDVLSDLPAPSSNVPSL	168-184	72.60%
		QDVLSDLPAPSSNVPSLL	168-185	72.60%
		QDVLSDLPAPSSNVPSLLA	168-186	72.60%
		QDVLSDLPAPSSNVPSLLAL	168-187	72.60%
		QDVLSDLPAPSSNVPSLLALL	168-188	72.60%
		QDVLSDLPAPSSNVPSLLALLR	168-189	72.60%
		QDVLSDLPAPSSNVPSLLALLRPL	168-191	72.60%
		QDVLSDLPAPSSNVPSLLALLRPLGLDATDLVTISGGH	168-205	72.60%
		QDVLSDLPAPSSNVPSLLALLRPLGLDATDLVTISGG-HTIGQAH	168-211	72.60%
		QDVLSDLPAPSSNVPSLLALLRPLGLDATDLVTISGGH-TIGQAHCSSFEDR	168-218	72.60%
		QYYVDLVNR	261-269	72.60%
		RGLSFDFYR	33-41	72.60%
		SATGPGEQQAPPNLTLRPSAFK	91-112	72.60%
		SCADILALAAR	131-141	72.60%
		SDQDLFTNDITRPIVER	275-291	72.60%
		SFDFYR	36-41	72.60%
DB2	Peroxidase 1 [Triticum aestivum]	SFDFYRR	36-42	72.60%
		SGGPDYRVPLGR	147-158	72.60%
		SQRDFFEQFGVSMGK	295-309	72.60%
		SVVVSGGPDYR	143-153	72.60%
		TIGQAHCSSFEDR	206-218	72.60%
		TISGGHTIGQAHCSSFEDR	200-218	72.60%

		TPNVFDNQYYVDLVN	254-268	72.60%
		TPNVFDNQYYVDLVNR	254-269	72.60%
		TPNVFDNQYYVDLVNREGLF	254-273	72.60%
		TSDLGEVR	317-234	72.60%
		TSDLGEVRR	317-235	72.60%
		TVLDVRTPNVFDNQYYVDLVNR	248-269	72.60%
		VFDNQYYVDLVNR	257-269	72.60%
		VLLDGSATGPGEQQAPPNLTLRPSAFK	86-112	72.60%
		VNDIRDR	114-120	72.60%
		VQDAVRK	55-61	72.60%
		VSDQDLFTNDITRPIVER	274-291	72.60%
		VVSCADILALAAR	129-141	72.60%
		VVSGGPDYR	145-153	72.60%
		VVSGGPDYRVPLGR	145-158	72.60%
		VVVSGGPDYR	144-153	72.60%
		YYVDLVNR	262-269	72.60%
DB2	Globulin 3 [Triticum aestivum]	AFVVPGLTDADGVGYVAQGEGVLTVIENGEK	182-212	30.60%
		AFVVPGLTDADGVGYVAQGEGVLTVIENGEKR	182-213	30.60%
		AKPLLASLSK	262-271	30.60%
		ALAQHDVR	367-374	30.60%
		ALRPFDEVSR	154-163	30.60%
		DTFNLLEQRPK	339-349	30.60%
		EVQEVFR	535-541	30.60%
		FHALAQHDVR	365-374	30.60%
		FNLLEQRPK	341-349	30.60%
		FQYFSAKPL	257-265	30.60%
		FQYFSAKPLLASLSK	257-271	30.60%
DB2	Globulin 3 [Triticum aestivum]	FSAKPLLASLSK	260-271	30.60%
		HALAQHDVR	366-374	30.60%
		IVAPAGSIMHLANTDGR	223-239	30.60%
		LRPFDEVSR	155-163	30.60%
		LYEADAR	357-363	30.60%
		NLLEQRPK	342-349	30.60%
		PAGSIMHLANTDGRR	226-240	30.60%

		PGLTDADGVGYVAQGEGVLTVIENGEKR	186-213	30.60%
		PLLASLSK	264-271	30.60%
		PLLASLSKR	264-272	30.60%
		QASEGDQGHHWPLPPFR	318-334	30.60%
		QGDVIVAPAGSIMHLAN	219-235	30.60%
		QGDVIVAPAGSIMHLANTDGR	219-239	30.60%
		QGDVIVAPAGSIMHLANTDGRR	219-240	30.60%
		QYFSAKPLLASLSK	258-271	30.60%
		RAFVVPGLTDADGVGYVAQGEGVLTVIENGEK	181-212	30.60%
		RLYEADAR	356-363	30.60%
		RPFDEVSR	156-163	30.60%
		RPYVFGPR	132-139	30.60%
		SAKPLLASLSK	261-271	30.60%
		SDHGFVKALRPFDEVSR	147-163	30.60%
		SFHALAQHDVR	364-374	30.60%
		TAPYLNTQSFK	386-396	30.60%
		TFNLLEQRPK	340-349	30.60%
		VAIMEVNPR	173-181	30.60%
		VAPAGSIMHLANTDGR	224-239	30.60%
		VAVANITPGSMTAPYLNTQSFK	375-396	30.60%
		YFSAKPLLASLSK	259-271	30.60%
