# Supplementary material for: A Mouse-Based Method to Monitor Wheat Allergens in Novel Wheat Lines and Varieties Created by Crossbreeding: Proof-of-Concept Using Durum and A. tauschii Wheats
Source: Int J Mol Sci. 2022 Jun 10;23(12):6505. doi: 10.3390/ijms23126505 (PMC9224339; doi:10.3390/ijms23126505)
Supplement: Supplementary file 1 [file ijms-23-06505-s001.zip › Supl_TableS4_DN1-DN3.pdf]

Table S4. Peptides sequences, positions, and coverage for wheat allergens identified in native tauschii wheat.

IgE-binding band	Protein	Sequence	Position	Percent coverage
DN1	Globulin-3A [Triticum aestivum]	AFVVPGLTDADGVGYVAQGEGVLTVIENGEK	182-212	33.50%
		AFVVPGLTDADGVGYVAQGEGVLTVIENGEKR	182-213	33.50%
		ALRPFDEVSR	154-163	33.50%
		DTFNLLEQR	339-347	33.50%
		DTFNLLEQRPK	339-349	33.50%
		EVQEVFR	535-541	33.50%
		FQYFSAK	257-263	33.50%
		FQYFSAKPLLASLSK	257-271	33.50%
		LAVVLEGEGEVEIVCPHLGR	397-416	33.50%
		LYEADAR	357-363	33.50%
		QASEGDQGHHWPLPPFR	318-334	33.50%
		QGDVIVAPAGSIMHLANTDGR	219-239	33.50%
		RPYVFGPR	132-139	33.50%
		SDHGFVK	147-153	33.50%
		SFHALAQHDVR	364-374	33.50%
		VAIMEVNPR	173-181	33.50%
		VAVANITPGSMTAPYLNTQSFK	375-396	33.50%
DN2	Dehydroascorbate reductase [Triticum aestivum]	AAVGHPDTLGDCPFSQR	8-24	67.50%
		AHGPYINGANISAVDLSLAPK	136-156	67.50%
		ALVDELQALEEHLK	122-135	67.50%
		ENLIAGWAPK	200-209	67.50%
		IFSTFVTFLK	102-111	67.50%
		LYHLQVALEHFK	157-168	67.50%
		VLLTLEEK	25-32	67.50%
		VPETLTSVHAYTEALFSR	172-189	67.50%
		WIADSDVITQVIEEK	69-83	67.50%
		WIADSDVITQVIEEKYPTPSLVTPPEYASVGSK	69-101	67.50%
		YPTPSLVTPPEYASVGSK	84-101	67.50%
		AAVGHPDTLGDCPFSQR	8-24	55.70%
		AHGPYINGANISAVDLSLAPK	136-156	55.70%
		ALVDELQALEEHLK	122-135	55.70%
		IFSTFVTFLK	102-111	55.70%
		LYHLQVALEHFK	157-168	55.70%
DN2	Dehydroascorbate reductase [Triticum aestivum]	VLLTLEEK	25-32	55.70%
		VPETLTSVHAYTEALFSR	172-189	55.70%
		YPTPSLVTPPEYASVGSK	84-101	55.70%

		AAVGHPDTLGDCPFSQR	8-24	67.50%
		AHGPYINGANISAVDLSLAPK	136-156	67.50%
		ALVDELQALEEHLK	122-135	67.50%
		IFSTFVTFLK	102-111	67.50%
		LYHLQVALEHFK	157-168	67.50%
		VLLTLEEK	25-32	67.50%
		VPETLTSVHAYTEALFSR	172-189	67.50%
		VPVYNGGDGK	59-68	67.50%
		WIADSDVITQVIEEK	69-83	67.50%
		YPTPSLVTPPEYASVGSK	84-101	67.50%
DN2	Class II chitinase [Triticum aestivum]	GAASDYCTPSAQWPCAPGKR	114-133	39.10%
		GPIQLSHNYNYGPAGR	138-153	39.10%
		NDGACQAK	42-49	39.10%
		RYCDILGVGYGDNLDCYNQRPFA	244-266	39.10%
		TAMWFWMTAQAPK	176-188	39.10%
		VPGFGVITNIINGGIECGHGQDSR	211-234	39.10%
		YCDILGVGYGDNLDCYNQR	245-263	39.10%
		YCDILGVGYGDNLDCYNQRPFA	245-266	39.10%
		GPIQLSHNYNYGPAGR	138-153	23.70%
		RYCDILGVGYGDNLDCYNQRPFA	244-266	23.70%
		VPGFGVITNIINGGIECGHGQDSR	211-234	23.70%
		YCDILGVGYGDNLDCYNQRPFA	245-266	23.70%
DN2	Endogenous alpha-amylase/subtilisin inhibitor	ADANYYVLPANR	15-26	62.80%
		AHGGGLTMAPGHGR	27-40	62.80%
		CPLFVSQEADGQR	42-54	62.80%
		DPPPVHDTDGNELR	1-14	62.80%
		DPPPVHDTDGNELRADANYYVLPANR	1-26	62.80%
		GGAWFLGATEPYHVVVFK	158-175	62.80%
		HVITGPVRDPSPSGR	107-121	62.80%
		LMACGDSCQDLGVFR	140-154	62.80%
DN2	Endogenous alpha-amylase/subtilisin inhibitor	RCPLFVSQEADGQR	41-54	62.80%
		RHVITGPVRDPSPSGR	106-121	62.80%
		YSGAEVHEYK	130-139	62.80%
		ADANYYVLPANR	15-26	40.00%
		CPLFVSQEADGQR	42-54	40.00%
		DPPPVHDTDGNELR	1-14	40.00%
		HVITGPVR	107-114	40.00%
		LMACGDSCQDLGVFR	140-154	40.00%
		YSGAEVHEYK	130-139	40.00%
		ADANYYVLPANR	15-26	56.10%

		AHGGGLTMAPGHGR	27-40	56.10%
		CPLFVSQEADGQR	42-54	56.10%
		CPLFVSQEADGQRDGLPVR	42-60	56.10%
		DPPPVHDTDGNELR	1-14	56.10%
		HVITGPVR	107-114	56.10%
		HVITGPVRDPSPSGR	107-121	56.10%
		LMACGDSCQDLGVFR	140-154	56.10%
		RCPLFVSQEADGQR	41-54	56.10%
		RHVITGPVR	106-114	56.10%
		RHVITGPVRDPSPSGR	106-121	56.10%
		YSGAEVHEYK	130-139	56.10%
DN2	Peroxidase 1 [Triticum aestivum]	DFFEQFGVSMGK	298-309	43.00%
		DSVVVSGGPDYR	142-153	43.00%
		DSVVVSGGPDYRVPLGR	142-158	43.00%
		EGLFVSDQDLFTNDITRPIVER	270-291	43.00%
		GAVVSCADILALAAR	127-141	43.00%
		GFVQDAVR	53-60	43.00%
		GLSFDFYR	34-41	43.00%
		KDIGLAAGLLR	61-71	43.00%
		LFPRPDPTINPPFLAR	219-234	43.00%
		PLGLDATDLVTISGGHTIGQAHCSSFEDR	190-218	43.00%
		TPNVFDNQYYVDLVNR	254-269	43.00%
		DFFEQFGVSMGK	298-309	43.30%
DN2	Peroxidase 1 [Triticum aestivum]	DSVVVSGGPDYR	142-153	43.30%
		EGLFVSDQDLFTNDITR	270-286	43.30%
		EGLFVSDQDLFTNDITRPIVER	270-291	43.30%
		GAVVSCADILALAAR	127-141	43.30%
		KDIGLAAGLLR	61-71	43.30%
		LFPRPDPTINPPFLAR	219-234	43.30%
		PDPTINPPFLAR	223-234	43.30%
		PLGLDATDLVTISGGHTIGQAHCSSFEDR	190-218	43.30%
		QDVLSDLPAPSSNVPSLLALLR	168-189	43.30%
		TPNVFDNQYYVDLVNR	254-269	43.30%
		DFFEQFGVSMGK	298-309	15.40%
		DSVVVSGGPDYR	142-153	15.40%
		GAVVSCADILALAAR	127-141	15.40%
		LFPRPDPTINPPFLAR	219-234	15.40%
DN2	Serpin-N3.2 [Triticum aestivum]	AEAQSVDFQTK	127-137	47.50%
		AFVEVNETGTEAAATTIAK	335-353	47.50%
		ATQSDDFYLLDGSSIQTPFMYSSEEQYISSSDGLK	192-226	47.50%

		DILPAGSIDNTTR	159-171	47.50%
		EDTSGVVLFIGHVVNPLLSS	379-398	47.50%
		LASAISSNPESTVNNAAFSPVSLHVALSLITAGAGGATR	23-61	47.50%
		LSAEPEFLEQHIPR	261-274	47.50%
		LVLGNALYFK	172-181	47.50%
		VAFANGVFVDASLQLKPSFQELAVCK	99-124	47.50%
		YKAEAQSVDFQTK	125-137	47.50%
DN2	Serpin-N3.2 [Triticum aestivum]	AFVEVNETGTEAAATTIAK	335-353	40.70%
		DILPAGSIDNTTR	159-171	40.70%
		EDTSGVVLFIGHVVNPLLSS	379-398	40.70%
		ISLGIEASDLLK	289-300	40.70%
		LASAISSNPESTVNNAAFSPVSLHVALSLITAGAGGATR	23-61	40.70%
		LSAEPEFLEQHIPR	261-274	40.70%
		LSIAHQTR	11-18	40.70%
		NQLAATLGEGEVEGLHALAEQVVQFVLADASNIGGPR	62-98	40.70%
		AEAQSVDFQTK	127-137	45.70%
DN2	Serpin-N3.2 [Triticum aestivum]	AFVEVNETGTEAAATTIAK	335-353	45.70%
		DILPAGSIDNTTR	159-171	45.70%
		EDTSGVVLFIGHVVNPLLSS	379-398	45.70%
		GLGLQLPFGAEADLSEMVDSPMAQNLYISSIFHK	301-334	45.70%
		ISLGIEASDLLK	289-300	45.70%
		LASAISSNPESTVNNAAFSPVSLHVALSLITAGAGGATR	23-61	45.70%
		LSAEPEFLEQHIPR	261-274	45.70%
		LSIAHQTR	11-18	45.70%
		LVLGNALYFK	172-181	45.70%
		YKAEAQSVDFQTK	125-137	45.70%
DN2	Gamma gliadin [Triticum aestivum]	DALLQQCSPVADMSFLR	60-76	11.70%
DN2	Serpin 3 [Triticum aestivum]	AETQSVDFQTK	127-137	49.60%
		CLGLQLPFSDEADFSEMVDSPMPQGLR	302-328	49.60%
		IKNILPSGSVDNTTK	157-171	49.60%
		ISFGIEASDLLK	290-301	49.60%
		LASTISSNPK	23-32	49.60%
		LSAEPDFLER	262-271	49.60%
		LVLANALYFK	172-181	49.60%
		MVPQQARPPSVMDFIADHPFLFLLR	355-379	49.60%
		NILPSGSVDNTTK	159-171	49.60%
		QFSMYILLPEAPGGLSSLAEK	241-261	49.60%
		SAASNAAFSPVSLHSALSLLAAGAGSATR	33-61	49.60%
		VSSVFHQAFVEVNEQGTEAAASTAIK	329-354	49.60%

		YKAETQSVDFQTK	125-137	49.60%
		DQLVATLGTGEVEGLHALAEQVVQFVLADASSAGGPR	62-98	36.80%
		ISFGIEASDLLK	290-301	36.80%
		LSIAHQTR	11-18	36.80%
		LVLANALYFK	172-181	36.80%
		MVPQQARPPSVMDFIADHPFLFLLR	355-379	36.80%
		SAASNAAFSPVSLHSALSLLAAGAGSATR	33-61	36.80%
		VSSVFHQAFVEVNEQGTEAAASTAIK	329-354	36.80%
		AETQSVDFQTK	127-137	46.60%
DN2	Serpin 3 [Triticum aestivum]	CLGLQLPFSDEADFSEMVDSPMPQGLR	302-328	46.60%
		IKNILPSGSVDNTTK	157-171	46.60%
		ISFGIEASDLLK	290-301	46.60%
		LASTISSNPK	23-32	46.60%
		LSAEPDFLER	262-271	46.60%
		LSIAHQTR	11-18	46.60%
		LVLANALYFK	172-181	46.60%
		NILPSGSVDNTTK	159-171	46.60%
		SAASNAAFSPVSLHSALSLLAAGAGSATR	33-61	46.60%
		VAFANGVFVDASLLLKPSFQELAVCK	99-124	46.60%
		VSSVFHQAFVEVNEQGTEAAASTAIK	329-354	46.60%
		YKAETQSVDFQTK	125-137	46.60%
DN3	Dimeric alpha-amylase inhibitor precursor, partial [Triticum aestivum]	CGALYSMLDSMYK	55-67	94.40%
		CGALYSMLDSMYKEHGAQEGQAGTGAFPR	55-83	94.40%
		DCCQQLAHISEWCR	41-54	94.40%
		DVAAYPDA	118-125	94.40%
		EHGAQEGQAGTGAFPR	68-83	94.40%
		EVVKLTAASITAVCR	87-101	94.40%
		LPIVVDASGDGAYVCK	102-117	94.40%
		LPIVVDASGDGAYVCKDVAAYPDA	102-125	94.40%
		LQCNGSQVPEAVLR	27-40	94.40%
		LTAASITAVCR	91-101	94.40%
		MSGPWMCYPGQAFQVPALPACR	1-22	94.40%
		SGPWMCYPGQAFQVPALPACR	2-22	94.40%
DN3	Unnamed protein product [Triticum aestivum]	DAVTYTEHAR	69-78	68.00%
		DNIQGITK	25-32	68.00%
		DNIQGITKPAIR	25-36	68.00%
		DNIQGITKPAIRR	25-37	68.00%
		IFLENVIR	61-68	68.00%
		ISGLIYEETR	47-56	68.00%
		ISGLIYEETRGVLK	47-60	68.00%

		KTVTAMDVVYALK	80-92	68.00%
		KTVTAMDVVYALKR	80-93	68.00%
DN3	Unnamed protein product [Triticum aestivum]	RISGLIYEETR	46-56	68.00%
		RISGLIYEETRGVLK	46-60	68.00%
		TLYGFGG	97-103	68.00%
		TVTAMDVVYALK	81-92	68.00%
		TVTAMDVVYALKR	81-93	68.00%
		VLRDNIQGITKPAIR	22-36	68.00%
