# Supplementary material for: A Mouse-Based Method to Monitor Wheat Allergens in Novel Wheat Lines and Varieties Created by Crossbreeding: Proof-of-Concept Using Durum and A. tauschii Wheats
Source: Int J Mol Sci. 2022 Jun 10;23(12):6505. doi: 10.3390/ijms23126505 (PMC9224339; doi:10.3390/ijms23126505)
Supplement: Supplementary file 1 [file ijms-23-06505-s001.zip › Supplementary Fig S1.pdf]

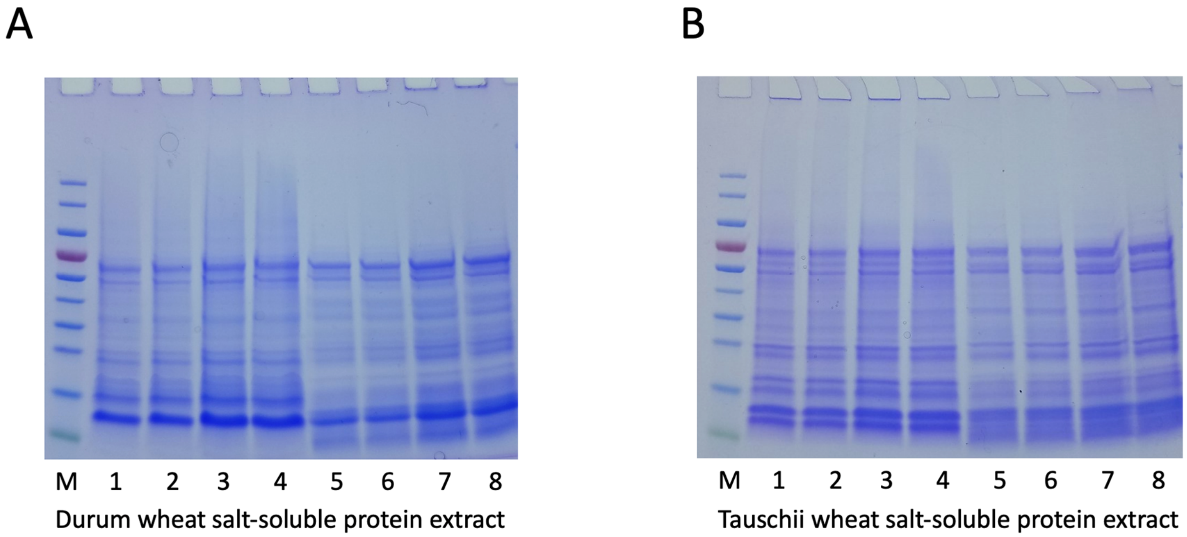

Figure S1. Sodium Dodecyl Sulfate Polyacrylamide Electrophoresis analysis of salt-soluble protein extracts used in this study. (A) Durum wheat salt-soluble protein extract; (B) Tauschii wheat salt-soluble protein extract. Lanes 1, 2 (30 microgram per lane), 3, 4 (50 microgram per lane): proteins were loaded in duplicates and electrophoresis was conducted under non-reducing conditions. Lanes 5, 6 (30 microgram per lane), 7, 8 (50 microgram per lane): proteins were loaded in duplicates and electrophoresis was conducted under boiling/reducing conditions. M = molecular mass markers (top to bottom): 180, 130, 95, 72, 55, 43, 34, 26, 17, 10 KDa.
